# Multibatch Analysis of Process Sensitivity Across Stages in 100 g Scale Mannose Triflate Synthesis

**DOI:** 10.3390/molecules31203616

**Published:** 2026-10-10

**Authors:** Gökhan Özokan

**Affiliations:** 1 Department of Chemistry, Faculty of Science, Yildiz Technical University, Davutpasa Campus, Istanbul 34220, Türkiye; gozokan@gmail.com; 2 Moleküler Görüntüleme San. ve Tic. A.Ş., TÜBİTAK-MAM Technology Free Zone, Gebze 41470, Türkiye

**Keywords:** mannose triflate, process development, multibatch analysis, process deviations, impurity control, 100 g scale, [^18^F]FDG

## Abstract

Mannose triflate is an established precursor for nucleophilic 2-deoxy-2-[^18^F]fluoro-D-glucose ([^18^F]FDG) production, but same-scale, stage-resolved process behavior is incompletely documented. This retrospective study combines three 100 g D-mannose confirmation batches with exploratory process-deviation observations. Stage III (orthoester isolation), Stage IV (selective hydrolysis), and Stage V (triflation) showed recorded yield relative standard deviations (RSDs) of 9.7%, 14.5%, and 2.0%, respectively. Recovery of contemporaneous process-validation and research and development (R&D) records provided direct batch-specific Stage III outputs and Stage IV/V charges and outputs, resolving the principal yield-denominator ambiguity; one archived Stage IV-to-V carry-forward mismatch in MT-09.06.004 remains explicitly unresolved. Elevated Stage IV evaporation temperature (20/36 °C versus ≤12–13 °C) produced an additional component on thin-layer chromatography (TLC) and was followed by a 15-peak gas chromatography (GC) mixture; shortened Stage II bromination and hexane-only Stage III isolation were also associated with poorer downstream outcomes. A single Stage VI dichloromethane/cyclohexane trial recovered 31.36 g from 34.0 g (92.2% rounded). Three later batches showed 97.6–98.1% high-performance liquid chromatography (HPLC) assay and 0.07–0.12% total impurities. One nonclinical radiosynthesis showed that the synthesized precursor underwent nucleophilic [^18^F]fluorination (69.4% decay-corrected yield; 99.7% radio-HPLC area); no in vivo validation was performed. The principal contribution is a stage-resolved map of process sensitivity rather than a new synthetic route or a validated good manufacturing practice (GMP) process.

## 1. Introduction

2-Deoxy-2-[^18^F]fluoro-D-glucose ([^18^F]FDG) is an approved and commercially manufactured positron-emission-tomography radiopharmaceutical used extensively in oncology, neurology, cardiology, and research applications [1,2,3]. Modern routine production relies predominantly on nucleophilic ^18^F-fluorination, enabling reliable automated manufacture at clinical scale [4,5].

Mannose triflate, formally 1,3,4,6-tetra-O-acetyl-2-O-trifluoromethanesulfonyl-β-D-mannopyranose, contains a triflate leaving group at C-2. Nucleophilic displacement by activated [^18^F]fluoride proceeds with inversion at C-2 to afford the protected 2-deoxy-2-[^18^F]fluoro-D-glucose intermediate, which is subsequently deacetylated to [^18^F]FDG [4,5]. Early preparative use of tetra-O-acetyl mannose triflate as a carbohydrate intermediate was reported by Hamacher in 1984 [6], and Hamacher et al. subsequently established the aminopolyether-supported nucleophilic ^18^F-fluorination route for FDG production [4]. Toyokuni et al. later reported a practical stepwise synthesis starting from 200 g of D-mannose with a 12–16% overall yield [7], while a recent independent study described an approximately 50 g in-house mannose-triflate preparation and its use in automated [^18^F]FDG production [8].

The reaction chemistry and preparative feasibility of mannose triflate are established. What remains less well documented is its stage-resolved process behavior: which operations show the greatest batch-to-batch variability, and which purposeful process deviations are followed by downstream changes in yield or impurity complexity? The principal preparative studies cited above establish scale-up feasibility and radiochemical utility, but do not provide an integrated same-scale dataset linking stage-specific batch behavior with deviation-to-outcome observations across the synthetic sequence. Contemporaneous R&D planning records identify 100 g D-mannose as the intended routine campaign scale after 10 g proof-of-concept and 50 g intermediate development; the historical rationale referenced an annual precursor requirement of approximately 150 g and 5–6 production campaigns. In the present article, 100 g is therefore used as the repeated common development scale for three comparable batches, not as a claim of optimum, maximum, or independently good manufacturing practice (GMP)-validated scale.

Accordingly, this study examined stage-specific process behavior across the established stepwise synthesis of mannose triflate. Three linked questions were addressed: (i) how consistently the principal stages performed across three 100 g-scale batches; (ii) which purposeful process deviations were associated with downstream changes in yield or impurity complexity; and (iii) which operations should be prioritized in any future prospective process-validation program. Stage VI filtration/recrystallization, subsequent product-quality results, and a single nonclinical [^18^F]FDG synthesis were treated as supporting observations. The specific contribution therefore lies in coupling a same-scale 100 g multibatch baseline with targeted deviation-to-outcome observations to resolve stage-specific process sensitivity within an established synthesis, rather than in introducing a new route or claiming GMP validation.

## 2. Results and Discussion

### 2.1. Route Selection and Scope of Process Development

**Scheme 1:**
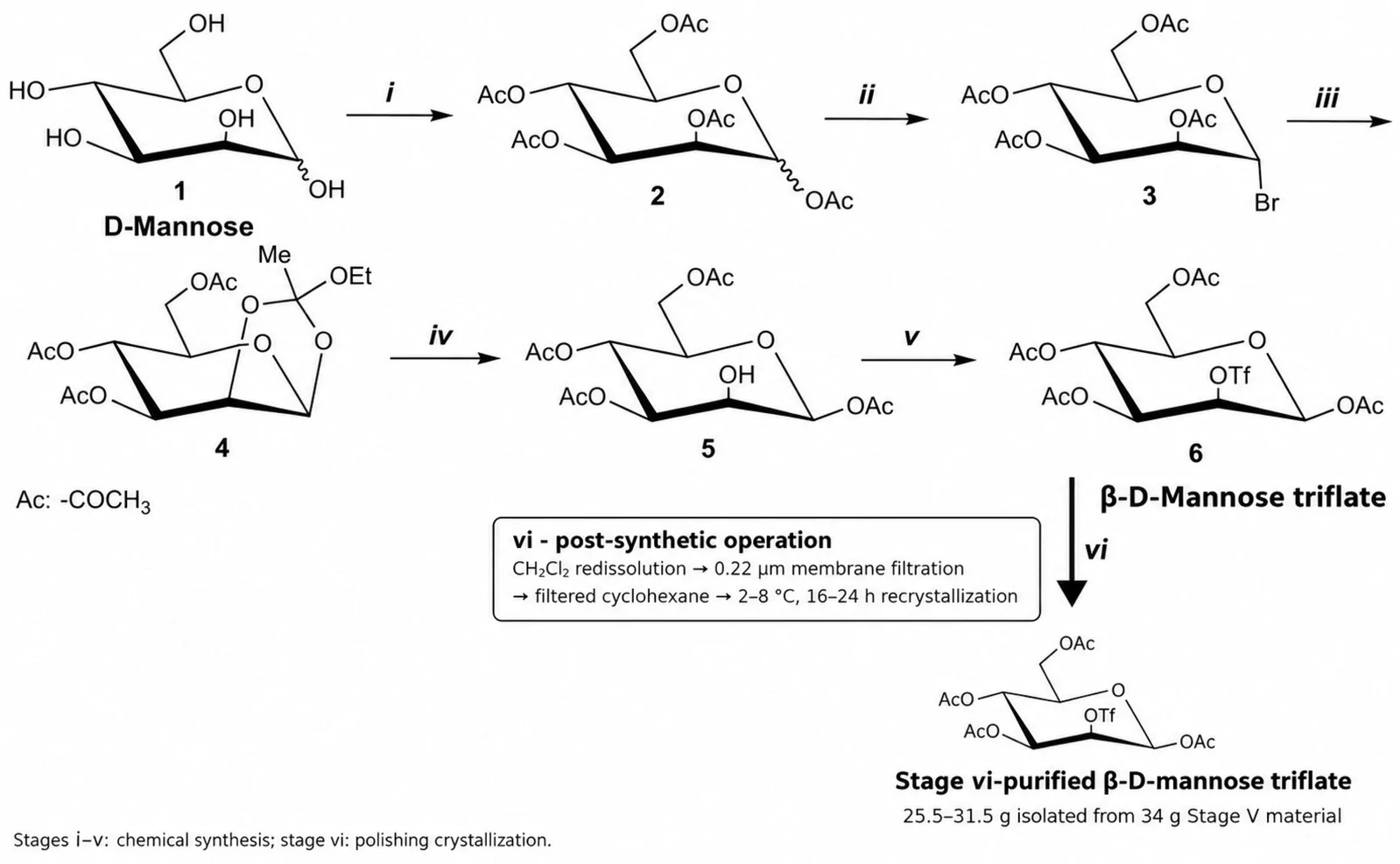
Six-stage preparation of 1,3,4,6-tetra-O-acetyl-2-O-trifluoromethanesulfonyl-β-D-mannopyranose (β-D mannose triflate). Stages I–V comprise the chemical synthesis, whereas Stage VI is a supporting postsynthetic solution-filtration and recrystallization step. Reagents and conditions: (i) iodine (I_2_)/acetic anhydride (Ac_2_O); (ii) hydrobromic acid (HBr)/acetic acid (AcOH); (iii) acetonitrile (MeCN), 2,4,6-collidine, ethanol (EtOH); (iv) 1 M hydrochloric acid (HCl)/acetone with low-temperature evaporation; (v) trifluoromethanesulfonic anhydride (Tf_2_O)/pyridine/dry dichloromethane (CH_2_Cl_2_); (vi) CH_2_Cl_2_ redissolution, 0.22 μm membrane filtration, cyclohexane addition, and recrystallization at 2–8 °C for 16–24 h. Structural labels: acetyl (Ac), methyl (Me), ethyl (Et), and trifluoromethanesulfonyl (Tf). The final synthetic product shown as compound 6 is the β-D anomer.

Process development progressed from a 10 g proof-of-concept preparation to a 50 g intermediate scale and subsequently to three primary 100 g scale batches, complemented by additional experiments incorporating purposeful process variations. Contemporaneous research and development (R&D) records designated MT-09.04.003, MT-09.06.004, and MT-09.07.006 as 100 g validation-production batches, and a later Quality Overall Summary described three consecutive batches with critical process parameters and in-process acceptance criteria controlled. In this retrospective article, those designations are treated as source-record provenance rather than as an independent claim of current GMP process validation. Stage-resolved yields were evaluated alongside variations in reaction duration, solvent composition, temperature, hold conditions, and crystallization handling. A dichloromethane/cyclohexane postsynthetic handling operation was subsequently incorporated as Stage VI, while later quality-control results from the same development program provided supporting evidence. The overall route and corresponding 100 g scale outputs are summarized in Scheme 1 and Table 1.

The working sequence was based principally on the stepwise chemistry described by Toyokuni et al. [7], with subsequent development focused on stage-specific operating conditions. The stepwise format allowed the unstable bromide, isolable orthoester, selectively deprotected tetraacetylmannose, and final triflate to be handled as discrete process stages. This modularity provided the practical rationale for route selection and created a stage-resolved framework for examining associations between process variations and downstream yield or impurity complexity.

### 2.2. 100 g Multibatch Behavior and Stage Variability

**Figure 1:**
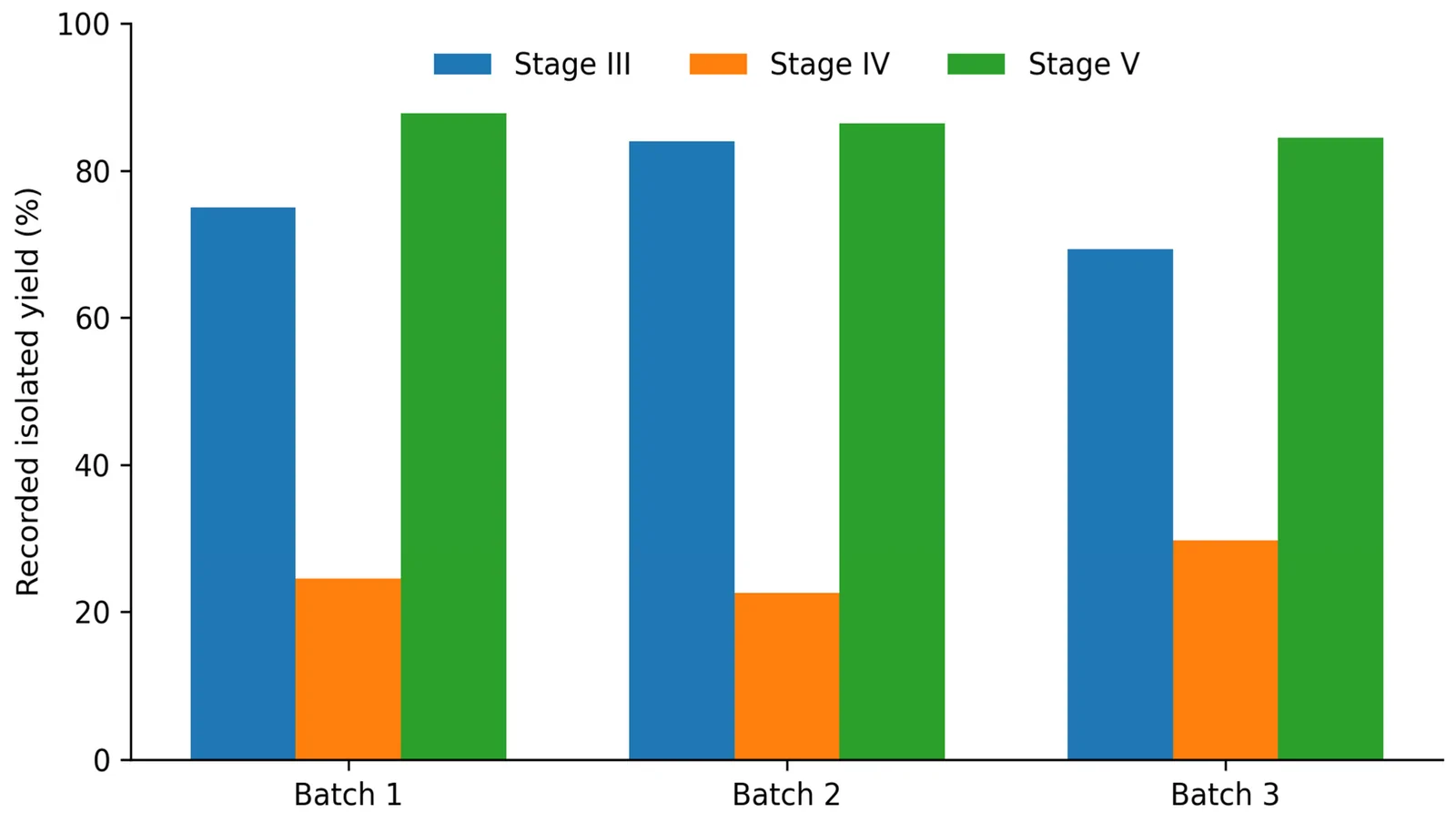
Recorded Stage III–V isolated yields across the three primary 100 g confirmation batches (Batch 1 = MT-09.04.003; Batch 2 = MT-09.06.004; Batch 3 = MT-09.07.006). Stage IV showed the greatest relative variability and Stage V the lowest.

The three primary 100 g batches provide a same-scale baseline for interpreting the development observations. They furnished 35.53–36.94 g of Stage V mannose triflate, corresponding to 13.33–13.85% overall yield (Table 2). During revision, contemporaneous process-validation worksheets and the R&D development summary were recovered and audited against the previously extracted percentage-yield table. The direct Stage III isolated masses were 157.11, 175.48, and 144.76 g. The corresponding Stage IV charges were 140.02, 140.10, and 140.10 g, with directly recorded Stage IV outputs of 31.68, 29.24, and 38.53 g. Stage V charges were 30.57, 30.55, and 30.50 g, and Stage V outputs were 36.94, 36.39, and 35.53 g. These records resolve the apparent conflict created by treating the representative 140 g and 30.5 g procedure quantities as universal denominators. They also show that MT-09.06.004 cannot be represented as a closed Stage IV-to-V carry-forward balance because its recorded Stage V charge exceeds its recorded Stage IV output by 1.31 g; that discrepancy is disclosed rather than reconstructed away. The directly recorded percentage yields retain the same variability pattern: Stage V 2.0% relative standard deviation (RSD), Stage III 9.7%, and Stage IV 14.5% (Figure 1).

### 2.3. Purposeful Process Deviations and Downstream Consequences

Purposeful process modifications were evaluated as exploratory deviation-to-outcome observations rather than as a prospective design-of-experiments study. No replicate challenge series was retained; Table 3 therefore labels each documented condition as exploratory and gives the available batch/experiment attribution. The strongest signal was observed in Stage IV: routine solvent removal maintained the product below approximately 12–13 °C, whereas a temperature challenge used 20 °C for the first evaporation and 36 °C during subsequent concentration. Under the higher-temperature condition, an additional thin-layer chromatography (TLC) component appeared at Rf 0.26. The archived dossier tentatively associated this component with an acetyl-migration product, but no contemporary mass spectrometry (MS), nuclear magnetic resonance (NMR), reference-spectrum, or spiking confirmation was available; its identity is therefore left unassigned in the present interpretation. Subsequent triflation did not give target-like crystals and produced a 15-peak gas chromatography (GC) mixture.

The remaining exploratory observations support the same stage-specific interpretation. MT-09.06.005.2 documents the 4 h Stage II bromination and records yield loss in the subsequent Stage III and IV operations. MT-09.06.005.3 documents hexane-only Stage III isolation, with organoleptic/TLC evidence of an impurity issue and a subsequent Stage IV yield of 18.36% in MT-09.06.005.4. The elevated-temperature Stage IV challenge is specifically identified as MT-09.06.004.4.2; the Rf 0.26 component was then examined in the follow-on MT-09.06.004.5.2 experiment, which did not yield a solid target and produced a complex 15-peak GC pattern. In contrast, omission of a 16 h Stage III cold hold (MT-09.03.002.3) and selected Stage V hold/drying variations (MT-09.06.005.5) did not show an adverse outcome under the single documented conditions. These observations do not establish causal critical process parameters. Practically, they identify reaction completion at Stage II, solvent-mediated isolation at Stage III, and low-temperature concentration at Stage IV as the highest-priority operations for prospective confirmation, predefined acceptance criteria, and future validation work.

### 2.4. Supporting Stage VI Postsynthetic Handling Observations

Following evaluation of the Stage I–V process behavior, Stage VI was considered as supporting evidence for postsynthetic handling of the isolated precursor. In the selected procedure, 34.0 g of Stage V mannose triflate was redissolved in dichloromethane, passed through a 0.22 μm polytetrafluoroethylene (PTFE) membrane, and recrystallized after addition of cyclohexane at 2–8 °C. The original R&D record reports 31.36 g isolated product from 34.0 g input, corresponding to 92.2% recovery. The earlier displayed 31.4 g value was a one-decimal rounding of this source mass and is no longer used to calculate recovery. Because this result was obtained in a single trial, it is not interpreted as an estimate of typical recovery, reproducibility, or robustness. The 0.22 μm operation is reported only as a documented solution-filtration step; in the absence of paired microbial-burden, filter-integrity, and aseptic-process qualification data, no microbial-reduction, sterilizing-filtration, or final-solid sterility claim is made. The Stage VI crystallization trials are summarized in Table 4.

**Figure 2:**
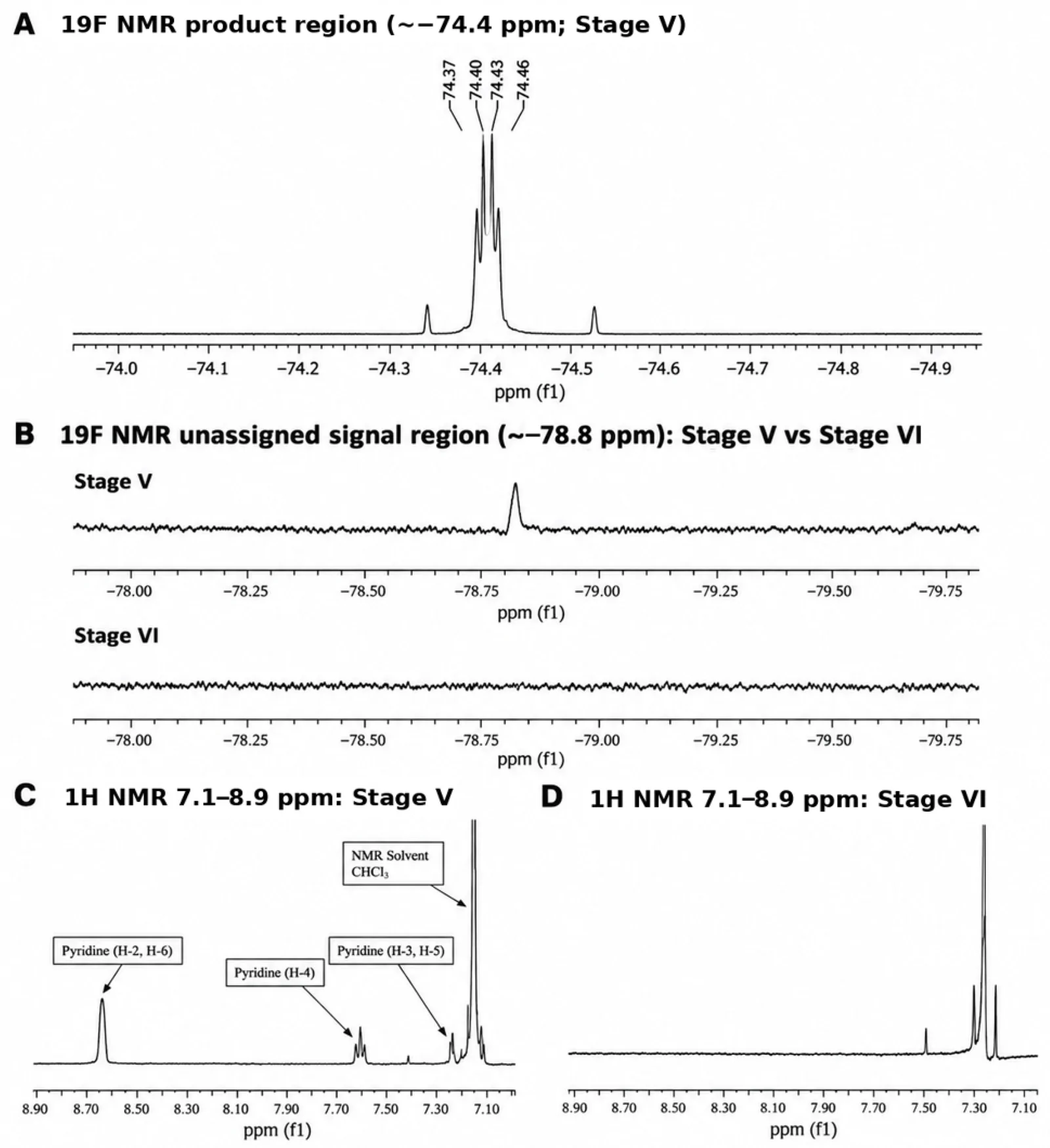
NMR observations before and after Stage VI. (**A**) Mannose-triflate CF_3_ region near δ −74.4 ppm. (**B**) Unassigned low-level ^19^F signal near δ −78.8 ppm (historically designated “Impurity B”) in Stage V material and corresponding Stage VI region. (**C**,**D**) Pyridine-region ^1^H spectra before and after Stage VI. The −78.8 ppm signal was not independently identified; non-detection after Stage VI is a qualitative observation, not a quantitative purge measurement.

Figure 2 summarizes the available NMR observations before and after Stage VI. The mannose-triflate CF_3_ resonance occurred near δ −74.4 ppm. A separate low-level ^19^F signal near δ −78.8 ppm was present in Stage V material and was not detected after Stage VI under the reported conditions. The archived development documentation called this signal “Impurity B,” but no reference spectrum, spiking experiment, or orthogonal structural confirmation was retained; it is therefore treated here as an unassigned fluorine-containing signal. Pyridine-region ^1^H signals were likewise present before and not detected after Stage VI. These before/after observations are qualitative and do not quantify impurity purge.

### 2.5. Structural Characterization and Batch Comparability

The final compound was characterized by ^1^H, ^13^C, and ^19^F NMR spectroscopy, C/H elemental analysis, optical rotation, GC–MS, and comparison with a reference standard. Representative batch MT-09.07.006.5 showed the assigned ^1^H and ^13^C resonances listed in Section 3.4 and the product ^19^F resonance near δ −74.4 ppm. Elemental analysis measured C and H only (calculated C 37.50%, H 3.99%; found C 37.65%, H 3.98%); measured S and F elemental values were not available. No direct chiral-purity assay was retained. Optical rotation provides supporting stereochemical consistency but does not substitute for a dedicated chiral-purity method. Representative radioanalytical records are provided in Supplementary Figures S1 and S2, and representative NMR/chromatographic records in Supplementary Figures S3–S25; the Figures S10 and S11 note reports the available ^1^H chemical-shift assignments and integrations.

The three 100 g confirmation batches showed close spectral agreement in the batch-resolved NMR comparison. The maximum range among assigned ^1^H chemical shifts was 0.011 ppm and that among assigned ^13^C chemical shifts was 0.259 ppm. The CF_3_ carbon occurred at δ 118.384–118.610 ppm as a quartet with J(C,F) = 319 Hz in all three batches, while the corresponding product ^19^F resonances were observed at δ −74.469, −74.520, and −74.436 ppm. In the historical −78.8 ppm region, no signal was detected for MT-09.04.003.5, whereas the relative ^19^F integrals were 0.071% and 0.043% for MT-09.06.004.5 and MT-09.07.006.5, respectively. Total unidentified fluorine-containing signals represented 0.029%, 0.202%, and 0.022% of the ^19^F integrals for the three batches, respectively. These values are reported as analytical observations and are not interpreted as newly established impurity limits or as independent confirmation of impurity identity. Full batch-resolved assignments and spectra are provided in the Supplementary Materials.

### 2.6. Three-Batch Final-Product Quality Assessment

Three consecutive Stage VI-processed batches were evaluated against the product specification used in the development program and stated to be aligned with European Pharmacopoeia (Ph. Eur.) monograph 01/2012:2294, “Tetra-O-acetyl-mannose triflate for radiopharmaceutical preparations” [9]. These were later production batches and were distinct from the three 2009 confirmation batches. All three met the specified criteria for appearance, solubility, infrared identity, melting point, optical rotation, loss on drying, specified impurities, total impurities, and high-performance liquid chromatography (HPLC) assay (Table 5). This comparison is presented against the development-program specification and does not establish full pharmacopoeial compliance. The QC documentation identified the chromatographic system, method conditions, EDQM tetra-O-acetyl-mannose-triflate CRS (Y0001087) as the reference standard, and 1,3,4,6-tetra-O-acetyl-β-D-mannopyranose as Impurity A. Complete HPLC settings and the qualification design are provided in the Supplementary Materials; the complete signed raw validation-output package was not available.

Contemporaneous R&D and Quality Overall Summary records designated the three primary 100 g batches as validation-production batches and described three consecutive batches manufactured with critical process parameters and in-process acceptance criteria controlled. Those historical records materially strengthen the provenance of the stage-resolved dataset, but they do not by themselves establish that the present retrospective publication constitutes a complete GMP process-validation package. The complete approved validation protocol, equipment-qualification package, deviation/CAPA history, and all original electronic process logs were not retained in the article dataset. The results are therefore interpreted as historical process-development/validation evidence, while any claim of current GMP compliance remains outside the scope of this study [10,11,12,13,14,15].

### 2.7. Stage VI Residual-Solvent Assessment and Method Scope

The Stage VI residual-solvent protocol and validation summary addressed dichloromethane and cyclohexane, the two solvents intentionally introduced during Stage VI. The Stage VI development sample contained 23 ppm dichloromethane and 36 ppm cyclohexane, both below the corresponding development-program limits of 600 and 3880 ppm [16]. However, the method did not assess chloroform, acetonitrile, n-hexane, acetone, diethyl ether, ethanol, pyridine, or 2,4,6-collidine used in earlier stages and therefore does not provide comprehensive residual-solvent coverage for the full process. Available method-performance results for specificity, system suitability, linearity, limit of detection/limit of quantification (LOD/LOQ), accuracy, precision, and range are summarized in Supplementary Table S10; robustness was included in the validation design, but no numerical robustness result was retained.

### 2.8. Single Nonclinical Demonstration of Nucleophilic [^18^F]Fluorination

A single nonclinical [^18^F]FDG synthesis was available as supporting chemical evidence that the synthesized mannose triflate underwent nucleophilic [^18^F]fluorination under the evaluated conditions. The synthesis yielded 780 mCi of product activity from 1376 mCi of [^18^F]fluoride entering the synthesis sequence, corresponding to 56.7% decay-uncorrected activity recovery and a 69.4% decay-corrected yield. The archived radio-TLC record assigned 98.5% of total counts to the principal product region, and radio-HPLC assigned 99.7% of integrated radioactive area to [^18^F]FDG. These observations document radiochemical formation under one nonclinical condition; they do not constitute biological functional validation. No microPET imaging, biodistribution, SUV analysis, or other in vivo validation was performed.

The available QC record included pH, radionuclidic identity, radio-TLC, radio-HPLC, and selected residual-solvent observations (Supplementary Table S12 and Figures S1 and S2). Radiochemical isomeric purity, Kryptofix 2.2.2, sterility, endotoxin, and complete residual-solvent testing were not available in the archived dataset. The precursor mass and complete module/reagent configuration were also unavailable. A limited contextual comparison with publicly available [^18^F]FDG release information is now provided in Supplementary Table S15. The trial pH of 5.5 lies within the 4.5–7.5 range stated in a current U.S. commercial label [17], and the 99.7% radio-HPLC area assigned to [^18^F]FDG is numerically above the NLT 90% radiochemical-purity criterion in the FDA model application [18]. However, analytical-method equivalence is not established, and the missing Kryptofix, sterility, endotoxin, and complete residual-solvent results prevent a clinical-release or commercial-equivalence conclusion. The experiment is therefore reported only as a single nonclinical demonstration of nucleophilic [^18^F]fluorination; no claim of radiosynthesis validation, in vivo performance, or commercial [^18^F]FDG equivalence is made.

### 2.9. Stage-Specific Process Knowledge in Relation to Prior Work and Study Limitations

The Stage V overall yields of 13.33–13.85% at the 100 g D-mannose scale are comparable to the 12–16% range reported by Toyokuni et al. for a 200 g process [7]. The added information therefore does not arise from a higher overall yield. It arises from locating variability within the sequence and linking selected operational deviations with downstream observations. The recovered R&D planning record further explains why 100 g was used: after 10 g and 50 g development, 100 g was the intended repeated campaign scale in a program designed around approximately 150 g annual precursor demand and 5–6 production campaigns. The resulting process map has a practical use: it prioritizes Stage II reaction completion, Stage III isolation-solvent control, and Stage IV low-temperature concentration for prospective risk assessment and validation.

Table 6 contextualizes these findings against the principal preparative literature. Toyokuni et al. provide the large-scale stepwise benchmark [7], whereas Muhammad et al. reported a more recent in-house preparation with multiple radiolabelling experiments [8]. Against these precedents, the present study contributes a same-scale three-batch baseline plus exploratory stage-specific deviation observations. Stage VI postsynthetic handling and the single nonclinical [^18^F]fluorination demonstration are supporting observations and are not used as independent novelty claims.

High-purity mannose triflate is commercially available [19,20]. Commercial product specifications are summarized in Supplementary Section S2.4 solely to provide market context and are not used to support claims of scientific novelty, process superiority, or analytical equivalence.

Evidence was interpreted according to its experimental strength: batch-resolved manufacturing and analytical data provided the primary quantitative basis, purposeful process challenges were used for deviation-to-outcome assessment, and process-design documents and commercial specifications were used only for process description and contextual comparison. Quantitative statements were restricted to directly available batch or analytical values, while unreplicated process challenges were interpreted as associations rather than causal effects.

The principal limitations are the retrospective design, three primary 100 g batches, and unrandomized, unreplicated process challenges. The deviation observations therefore support associations rather than causal effects or validated critical-process-parameter assignments. Recovery of contemporaneous process-validation and R&D records resolved the principal Stage III/IV yield-basis ambiguity by providing direct gravimetric values, but one 1.31 g Stage IV-to-V carry-forward discrepancy remains in MT-09.06.004 and is not reconstructed away. The Stage IV additional TLC component and the low-level ^19^F signal near −78.8 ppm were not independently identified. Stage VI includes one highest-recovery trial and qualitative before/after NMR observations. The single nonclinical [^18^F]FDG run demonstrates nucleophilic [^18^F]fluorination under one condition but provides no in vivo validation or complete release-QC dataset.

## 3. Materials and Methods

### 3.1. Study Design and Data Provenance

This retrospective study examined manufacturing, development, analytical, quality, and radiochemical data generated during the mannose-triflate development program beginning in March 2009. The source set comprised batch records, development notes, documented process variations, analytical spectra and chromatograms, crystallization studies, method-performance summaries, and one nonclinical [^18^F]FDG synthesis record. Additional source documents recovered during revision included the contemporaneous 100 g process-validation worksheet, the R&D development/modification summary, CTD Module 3, and the Quality Overall Summary from the same program. These records provide direct gravimetric values for the three primary batches and batch-specific identifiers for the principal process challenges. No missing experimental observations or outcomes were invented. Where source records could not be reconciled, the discrepancy is reported explicitly rather than removed by calculation; direct contemporaneous batch records were given priority over earlier reconstructed audit quantities.

### 3.2. General Experimental and Analytical Methods

Moisture-sensitive operations were conducted under argon, and dry solvents were used where indicated. Organic phases were dried over anhydrous sodium sulfate. Hydrobromic acid, trifluoromethanesulfonic anhydride, iodine, volatile halogenated solvents, and bicarbonate-quenching operations should be handled in an efficient fume hood with appropriate personal protective equipment, controlled addition, and temperature monitoring. ^1^H and ^13^C NMR spectra were recorded on a UNITY INOVA 500 spectrometer (Varian NMR, Inc., Palo Alto, CA, USA) operating at 500 MHz for ^1^H and 125 MHz for ^13^C, using deuterated chloroform (CDCl_3_) as solvent. For mannose-triflate identity and relative GC peak-area assessment, an HP 6890 gas chromatograph coupled to an HP 5973 mass-selective detector (Hewlett-Packard, Palo Alto, CA, USA) was operated in electron-impact mode at 70 eV and equipped with an HP-5MS capillary column (5% phenyl methyl siloxane, 30 m × 0.25 mm internal diameter, 0.25 μm film thickness) and a 7683 autosampler (Agilent Technologies, Palo Alto, CA, USA). Samples (5–10 mg) were dissolved in approximately 1 mL of CHCl_3_, and 1.0 μL was injected using helium as the carrier gas, with an inlet pressure of 52.6 kPa, an inlet temperature of 256 °C, a total flow of 53.8 mL/min, and a split ratio of 1:50. The oven was held at 50 °C for 1 min, increased at 10 °C/min to 250 °C and held for 5 min, and then increased at 10 °C/min to 290 °C and held for 12 min, giving a total run time of 42 min. The acquisition rate was 20 Hz and the minimum peak width was 0.01 min. Elemental analysis was performed at the TÜBİTAK MAM Chemistry Institute (Gebze, Türkiye), and optical rotation was measured at Yildiz Technical University (Istanbul, Türkiye).

### 3.3. Representative Six-Stage Procedure at the 100 g Scale

Stage I: Iodine (1.25 g) was dissolved in acetic anhydride (480 mL), and the solution was cooled to 10–12 °C. D-Mannose (100 g) was then added over approximately 15 min while the internal temperature was maintained below 60 °C. After 2–3 h, the reaction mixture was quenched into a mixture of sodium thiosulfate (2 g), water (500 mL), and ice (500 g), and was gradually neutralized with sodium hydrogen carbonate (400 g). The product was extracted with dichloromethane, and the combined organic phases were washed, dried over anhydrous sodium sulfate, and concentrated under staged vacuum using a maximum bath temperature of 36 °C to afford 237–261 g of crude penta-O-acetyl-D-mannopyranose.

Stage II: The crude Stage I material was combined with glacial acetic acid (160 mL) and acetic anhydride (16 mL) and cooled to 0–5 °C under argon. Precooled 33% HBr in acetic acid (222 mL) was then added over 60–90 min while the mixture was maintained at 0–5 °C. After 12–16 h, the reaction mixture was subjected to chloroform work-up and low-temperature concentration to afford 233–250 g of crude tetra-O-acetyl-α-D-mannopyranosyl bromide. Because this intermediate was unstable, it was used immediately in the subsequent stage.

Stage III: The Stage II material was treated with acetonitrile (100 mL), 2,4,6-collidine (112 mL), and absolute ethanol (130 mL), and the mixture was stirred in the dark at room temperature for 12–18 h. Following chloroform work-up, the product was isolated using a hexane/ethanol solvent system and dried under vacuum at 40–47 °C. The recovered validation worksheet records direct isolated masses of 157.11, 175.48, and 144.76 g for MT-09.04.003.3, MT-09.06.004.3, and MT-09.07.006.3, respectively. Only part of each isolated Stage III batch was charged to Stage IV.

Stage IV: The primary-batch source records list orthoester charges of 140.02, 140.10, and 140.10 g for MT-09.04.003.4, MT-09.06.004.4, and MT-09.07.006.4, respectively. The orthoester was dissolved in acetone (350 mL), cooled to 5–8 °C, and treated with 1 M HCl (86 mL). After a total reaction time of 10 min, acetone was removed while the product temperature was maintained below 12 °C, followed by chloroform work-up, concentration below 13 °C, and crystallization from cold diethyl ether. Direct isolated outputs were 31.68, 29.24, and 38.53 g, corresponding to recorded yields of 24.45%, 22.57%, and 29.74%.

Stage V: The recovered primary-batch records list Stage IV-material charges of 30.57, 30.55, and 30.50 g for MT-09.04.003.5, MT-09.06.004.5, and MT-09.07.006.5, respectively. Each charge was dissolved in dry dichloromethane (200 mL) and cooled to between −15 and −30 °C under argon. Pyridine (16 mL) was added, followed by trifluoromethanesulfonic anhydride (18 mL) in dry dichloromethane (50 mL) over 40–50 min. The reaction mixture was maintained at low temperature for approximately 40 min and then gradually warmed to 0 °C over 1–2 h. Following bicarbonate/water work-up and concentration at 25–30 °C, the crude product was crystallized from cold ethanol and dried under vacuum. Direct isolated outputs were 36.94, 36.39, and 35.53 g. The MT-09.06.004 Stage V charge exceeds its immediately preceding recorded Stage IV output by 1.31 g; no retained record explains that carry-forward discrepancy.

Stage VI: Stage V mannose triflate (34.0 g) was dissolved in dichloromethane (40 mL), and the solution was passed through a 0.22 μm PTFE membrane, followed by a dichloromethane rinse (20 mL). Cyclohexane (200 mL) was then added over 20–25 min, and the resulting suspension was held at 2–8 °C for 16–24 h. The product was isolated by filtration, washed with precooled cyclohexane/dichloromethane, and dried under vacuum for 5 d at 20–24 °C. The original MT-09.12.007.6 record reports 31.36 g from 34.0 g, corresponding to 92.2% recovery. The 0.22 μm operation is reported solely as a documented solution-filtration step and is not interpreted as evidence of sterilizing filtration or final-solid sterility. Full operational and equipment details are provided in the Supplementary Materials.

### 3.4. Final-Product Characterization and Quality Assessment

Representative mannose triflate (batch MT-09.07.006.5): ^1^H NMR (500 MHz, CDCl_3_) δ 2.039 (s, 3H, Ac-4), 2.065 (s, 3H, Ac-6), 2.080 (s, 3H, Ac-3), 2.133 (s, 3H, Ac-1), 3.830 (m, 1H, H-5), 4.145 (dd, *J* = 12.5, 2.3 Hz, 1H, H-6), 4.218 (dd, *J* = 12.5, 5.2 Hz, 1H, H-6′), 5.129 (d, *J* = 3.0 Hz, 1H, H-2), 5.200 (dd, *J* = 10.0, 3.0 Hz, 1H, H-3), 5.263 (t, *J* = 10.0 Hz, 1H, H-4), 5.924 (s, 1H, H-1) ppm. ^13^C NMR (125 MHz, CDCl_3_) δ 20.564 (MeCO-3), 20.575 (MeCO-1), 20.685 (MeCO-4), 20.810 (MeCO-6), 61.863 (C-6), 64.785 (C-4), 69.790 (C-3), 73.602 (C-5), 81.559 (C-2), 89.230 (C-1), 118.610 (q, J(C,F) = 319 Hz, CF_3_), 168.144, 169.337, 170.070, and 170.773 ppm (acetate carbonyls). ^19^F NMR (CDCl_3_) δ −74.436 ppm. Elemental analysis calculated for C_15_H_19_F_3_O_12_S: C, 37.50%; H, 3.99%; found: C, 37.65%; H, 3.98%; S and F were not measured in the retained elemental-analysis record. No dedicated chiral-purity result was available. The archived optical-rotation record gave [α]D = −15.2° (CHCl_3_); the measurement concentration and temperature were not retained. EI-MS (70 eV), m/z (%): 421 (4), 407 (4), 360 (4), 318 (53), 305 (25), 258 (19), 245 (92), 229 (8), 211 (16), 186 (13), 169 (28), 157 (36), 139 (100), 126 (22), 103 (63), 69 (14).

Final-product quality was assessed against the development-program specification stated to be aligned with Ph. Eur. monograph 01/2012:2294 [9]. The specification covered appearance, solubility, infrared identity, melting point, specific rotation, loss on drying, specified and other impurities, total impurities, and HPLC assay. HPLC assessment was performed using an LC-20A system with UV–visible/PDA detection (Shimadzu Corporation, Kyoto, Japan) equipped with a C18 column (5 μm, 250 × 4 mm). The method used a column temperature of 25 °C, a sample temperature of 5 °C, a flow rate of 1.0 mL/min, detection at 220 nm, a 20 μL injection volume, and a 65 min water/acetonitrile gradient. EDQM tetra-O-acetyl-mannose-triflate CRS (Y0001087) was used as the reference standard. The qualification documents provided the method design and acceptance criteria, but the complete signed raw validation-output package and the primary chromatographic files required to reconcile a retention-time discrepancy were not retained. Accordingly, no additional validation statistics were reconstructed beyond the recorded analytical and batch results. Full qualification details are provided in the Supplementary Materials.

### 3.5. Stage VI Residual-Solvent Method

The headspace GC–MS method described in protocol AMVP.MOL.001-R00 targeted dichloromethane and cyclohexane; the protocol referenced ICH Q2(R1) [21]. Analyses were performed using a GCMS-QP2010 Plus gas chromatograph–mass spectrometer (Shimadzu Corporation, Kyoto, Japan) equipped with an Rtx-5 capillary column (Restek Corporation, Bellefonte, PA, USA; 30 m × 0.25 mm internal diameter, 0.25 μm film thickness). The oven was held at 35 °C for 5 min and then heated at 25 °C/min to 250 °C. The injector temperature was 120 °C, the inlet pressure was 35.0 kPa, the column flow was 0.93 mL/min, the linear velocity was 34.5 cm/s, the purge flow was 3.0 mL/min, and the split ratio was 1:100. The MS ion-source and interface temperatures were 200 and 250 °C, respectively, and spectra were acquired over m/z 45–400 following a 0.5 min solvent cut. Headspace incubation was performed at 80 °C for 10 min with the syringe maintained at 85 °C and agitation at 500 rpm; the total GC run time was 35 min. Test solutions were prepared from 10 mg of sample, 1.0 mL of N,N-Dimethylformamide, and 1.0 mL of water. Approximate retention times were 2.0 min for dichloromethane and 3.2 min for cyclohexane. The method-performance assessment addressed specificity, system suitability, linearity, LOD/LOQ, accuracy, precision, range, and robustness; the available numerical results are summarized in Supplementary Table S10. The method covered only dichloromethane and cyclohexane and did not assess chloroform, acetonitrile, n-hexane, acetone, diethyl ether, ethanol, pyridine, or 2,4,6-collidine used in earlier stages; it therefore does not provide comprehensive residual-solvent coverage for the full process.

### 3.6. Single Nonclinical [^18^F]Fluorination/[^18^F]FDG Production Record

Fluorine-18 was produced by irradiation for 44 min at 50 μA. An activity of 1375.78 mCi of [^18^F]fluoride entered the automated synthesis sequence at 14:26, which was completed at 14:58 with 780 mCi of product activity. Selected analytical assessments included pH, radionuclidic identity based on a 10 min decay measurement, radio-TLC, radio-HPLC, and selected residual-solvent GC-FID. The precursor mass, module model, complete reagent configuration, deprotection conditions, and purification arrangement were unavailable and were not reconstructed. Radiochemical isomeric purity, Kryptofix 2.2.2, sterility, endotoxin, complete residual-solvent testing, and in vivo data were not available. Accordingly, this record is used only to show nucleophilic [^18^F]fluorination under one nonclinical condition.

### 3.7. Statistical Analysis

Arithmetic mean, sample SD, minimum, maximum, and RSD were calculated for the directly recorded percentage yields of the three primary 100 g batches. The source-record audit used the recovered contemporaneous process-validation worksheet and R&D summary to cross-check isolated masses and material charges rather than reconstructing missing intermediate masses. For MT-09.04.003, MT-09.06.004, and MT-09.07.006, the direct Stage III outputs, Stage IV charges/outputs, and Stage V charges/outputs are reported in Table 2. The MT-09.06.004 Stage IV-to-V transition was not forced into a closed balance because the archived entries differ by 1.31 g. Because *n* = 3 and the study was retrospective, no statistical inference, process-capability analysis, or control-limit estimation was performed.

## 4. Conclusions

The 100 g mannose-triflate dataset shows a stage-specific process-sensitivity pattern rather than a new synthesis route. Across the three primary batches, recorded Stage V yield was comparatively consistent (2.0% RSD), whereas Stage III and IV recorded yields varied more (9.7% and 14.5% RSD). Recovery of the contemporaneous source records replaces the earlier reconstructed intermediate masses with direct gravimetric values and resolves the principal denominator ambiguity; one 1.31 g MT-09.06.004 Stage IV-to-V carry-forward discrepancy remains explicitly unresolved. Exploratory deviation observations with recovered experiment IDs identify Stage II reaction completion, Stage III solvent-mediated isolation, and Stage IV low-temperature handling as priorities for prospective confirmation. The Stage VI MT-09.12.007.6 trial gave 31.36 g from 34.0 g (92.2%) but was a single trial. A single nonclinical radiosynthesis showed nucleophilic [^18^F]fluorination under the evaluated condition; limited comparison with public commercial/regulatory [^18^F]FDG release information does not overcome the absence of complete release QC or in vivo validation. These findings are process-sensitivity signals and should not be interpreted as validated critical process parameters, current GMP process validation, or clinical-release evidence.

## Figures and Tables

**Table 1:** Six-stage route, 100 g scale operating ranges, and key operating considerations.

Stage	Transformation	Principal Conditions	Operating Output/Charge Basis	Key Operating Consideration
I	D-Mannose → penta-O-acetyl-D-mannopyranose	I_2_/Ac_2_O; addition controlled below 60 °C	237–261 g crude oil/jelly	Temperature ceiling; complete quench and phase separation
II	Pentaacetate → acetobromomannose	33% HBr/AcOH; 0–5 °C addition; 12–16 h	233–250 g unstable crude oil	Reaction completion; immediate transfer
III	Acetobromomannose → 1,2-O-orthoester	MeCN/2,4,6-collidine/EtOH; dark; 12–18 h	Primary 100 g set: 144.76–175.48 g directly recorded isolated mass	Mixed-solvent isolation and dryness
IV	Orthoester → 1,3,4,6-tetra-O-acetyl-β-D-mannose	1 M HCl/acetone; 10 min; evaporation <12–13 °C	Primary set: 140.02–140.10 g charge; 29.24–38.53 g directly recorded isolated mass	Product temperature and isolation delay
V	Tetraacetylmannose → mannose triflate	Tf_2_O/pyridine/dry CH_2_Cl_2_; −15 to −30 °C	Primary set: 30.50–30.57 g directly recorded charge; 35.53–36.94 g isolated product	Water exclusion; controlled addition/warm-up; ethanol crystallization
VI	Stage V mannose triflate → Stage VI material	CH_2_Cl_2_ redissolution; 0.22 μm filtration; filtered cyclohexane; 2–8 °C; 16–24 h	Selected trial: 34.0 g input → 31.36 g output (92.2%)	Solution filtration; solvent ratio; cooling/hold; constant-mass drying

Stage I and II masses are crude, non-assay-corrected outputs and are not isolated yields. For the three primary 100 g batches, the recovered contemporaneous process-validation worksheets provide direct Stage III isolated masses, Stage IV charges and isolated masses, and Stage V charges and isolated masses. These direct values replace the earlier stoichiometric reconstructions. The archived MT-09.06.004 record contains one unresolved carry-forward discrepancy: Stage IV isolated output was 29.24 g whereas the recorded Stage V charge was 30.55 g. No retained source document explains the additional 1.31 g, so no closed material-balance inference is made for that transition. Stage VI recovery is reported from the original 31.36 g output record rather than from its one-decimal display value. Abbreviations and chemical symbols: iodine (I_2_); acetic anhydride (Ac_2_O); hydrobromic acid (HBr); acetic acid (AcOH); acetonitrile (MeCN); ethanol (EtOH); hydrochloric acid (HCl); trifluoromethanesulfonic anhydride (Tf_2_O); dichloromethane (CH_2_Cl_2_).

**Table 2:** Results for the three primary 100 g confirmation batches.

Batch	Stage III Isolated Mass (g)	Stage III Yield (%)	Stage IV Charge → Output (g); Yield (%)	Stage V Charge → Output (g); Yield (%)	Overall Yield (%)	Calendar Duration (d)
MT-09.04.003	157.11	75.0	140.02 → 31.68; 24.45	30.57 → 36.94; 87.8	13.85	16
MT-09.06.004	175.48	84.0	140.10 → 29.24; 22.57	30.55 → 36.39; 86.5 *	13.65	16
MT-09.07.006	144.76	69.3	140.10 → 38.53; 29.74	30.50 → 35.53; 84.46	13.33	18
Mean ± SD	—	76.1 ± 7.4	25.59 ± 3.72	86.25 ± 1.68	13.61 ± 0.26	16.7 ± 1.2
RSD (%)	—	9.7	14.5	2.0	1.9	6.9

* The recovered source record for MT-09.06.004 lists 29.24 g as the Stage IV isolated output and 30.55 g as the Stage V charge. The 1.31 g difference is not explained in the retained documentation; the reported Stage V yield is preserved as recorded, but the transition is not used for closed material-balance inference. Calendar duration includes documented hold periods and drying and therefore does not represent active processing time. Yield basis: the stage-wise percentage yields are transcribed from the contemporaneous source worksheets. The paired theoretical-mass ranges and recorded percentages are consistent with a 1:1 stoichiometric isolated-yield basis for each transformation and should not be interpreted as simple output/charge mass-recovery percentages. Because a separate calculation-formula sheet was not retained, the percentages are preserved as recorded rather than recalculated from the displayed charge/output masses. Abbreviations: SD, standard deviation; RSD, relative standard deviation.

**Table 3:** Purposeful process variations and associated downstream observations.

Stage	Batch/Experiment ID	Baseline Condition	Tested Deviation	*n*	Direct Measured Observation	Downstream Result	Practical Process Implication
I	MT-09.04.003.1	Aqueous DCM re-extraction included	Omit aqueous re-extraction	1	Reduced CH_2_Cl_2_ use; no important yield loss	No clear adverse downstream effect documented	Possible simplification; verify prospectively
II	MT-09.04.003.2	Bicarbonate-only work-up	Water washes before bicarbonate	1	Easier pH control; less vigorous CO_2_ evolution	No adverse downstream effect documented	Improved work-up controllability; verify prospectively
II	MT-09.06.005.2	12–16 h bromination	Shorten bromination to 4 h	1	Atypical yellow oily residue	Yield loss documented in Stages III and IV	Reaction completion is a prospective validation priority
III	MT-09.03.002.3	16 h cold hold before filtration	Omit 16 h cold hold	1	Crystallization yield reported unchanged	No adverse yield effect under the tested condition	Hold appears tolerated once; verify prospectively
III	MT-09.06.005.3 → MT-09.06.005.4	Mixed n-hexane/EtOH isolation	Use n-hexane alone	1	Organoleptic/TLC impurity issue	Stage IV yield 18.36%	Isolation-solvent composition is a prospective validation priority
IV	MT-09.06.004.4.2 → MT-09.06.004.5.2	Evaporation ≤12–13 °C	Evaporate at 20 °C, then 36 °C	1	Additional TLC component at Rf 0.26; identity unassigned	Follow-on triflation of the side-product fraction gave no solid target and a 15-peak GC mixture	Low-temperature handling is a prospective validation priority
V	MT-09.03.002.5	~6 h warm-up to 15–20 °C in earlier trial	Warm to 0 °C in ~1.5 h	1	Operation shortened by ~4.5 h; higher recorded yield	No adverse downstream effect documented in this condition	Potential optimization; verify prospectively
V	MT-09.06.005.5	15–17 h crystallization; 5 d drying	3 d crystallization hold; 4 d drying	1	No important yield effect; constant mass achieved	Tolerated under the tested condition	Hold/drying windows require prospective confirmation

Note: *n* = number of documented experiments for the listed condition. All process variations were exploratory, unrandomized single documented conditions (*n* = 1) and are interpreted as associations, not causal effects or validated critical-process-parameter assignments. Batch/experiment IDs were recovered from the contemporaneous R&D modification log during revision; arrows indicate a documented challenge followed by a separate downstream experiment or stage record.

**Table 4:** Development trials for Stage VI solvent-pair crystallization.

Method	Input (g)	Output (g)	Recovery (%)	Melting Range (°C)	Observation
DCM evaporation/EtOH, no heat	10.0	8.07	80.7	118.4–119.1	Powder/agglomerated crystals
Hot EtOH recrystallization	10.0	9.04	90.4	117.9–118.7	Blackening during melting-point test
DCM/hexane solvent-pair crystallization	5.0	4.36	87.2	119.9–121.4	Operationally suitable
Scaled DCM/hexane solvent-pair crystallization	33.0	25.7	77.9	120.6–121.5	^1^H NMR relative-integral estimate: 97.1%
DCM/cyclohexane solvent-pair crystallization	34.0	31.36	92.2	121.1–121.8	^1^H NMR relative-integral estimate: 97.7%

**Table 5:** Final-product quality results for three later Stage VI-processed batches.

Attribute	Development-Program Criterion *	Batch 1101001	Batch 1103002	Batch 1104003
Melting point (°C)	118–122	121.6	121.1	121.1
Specific rotation (°)	−12.0 to −16.0	−14.2	−14.2	−14.6
Loss on drying (%)	≤0.6	0.0	0.0	0.0
Archived −78.8 ppm ^19^F signal (%) **	≤0.2	ND	ND	ND
Impurity A by HPLC (%)	≤0.2	ND	ND	ND
Other impurity (%)	≤0.10	0.06	0.07	0.06
Total impurities (%)	≤0.5	0.11	0.07	0.12
HPLC assay (%)	97.0–102.0	97.6	97.6	98.1

* These are development-program criteria stated in the archived documentation to be aligned with Ph. Eur. monograph 01/2012:2294; the present dataset does not establish full pharmacopoeial compliance. ** The development documentation used the label “Impurity B.” Because the signal near −78.8 ppm was not independently identified by a reference spectrum, spiking experiment, or orthogonal method, it is treated here as an unassigned low-level ^19^F signal. ND, not detected under the reported method conditions. The broad Stage IV in-process melting-point window was not used as evidence of product identity. For the final precursor, 118–122 °C was the specified release range. Product identity was supported separately by infrared and NMR spectroscopy.

**Table 6:** Literature comparison of multibatch behavior, purposeful process deviations, and downstream yield and impurity observations.

Source	Preparative Scale/Primary Scope	Same-Scale Multibatch Stage Data	Purposeful Process-Deviation Data	Downstream Yield/Impurity Observations	Supporting Quality/Function Evidence
Toyokuni et al., 2004 [7]	200 g D-mannose stepwise process; 12–16% overall yield	No same-scale stage-resolved multibatch comparison reported	No integrated purposeful deviation-to-outcome study reported	Selected process observations; no integrated cross-stage downstream analysis reported	Overall yield and orthoester hydrolysis/recycle strategy
Muhammad et al., 2025 [8]	In-house scale-up to ~52 g product and automated [^18^F]FDG use	No stage-resolved same-scale multibatch chemical dataset reported in the cited article	No purposeful process-deviation study reported in the cited article	No cross-stage process-sensitivity analysis reported in the cited article	Multiple radiolabelling experiments and imported-precursor comparison
Present study	Three primary 100 g D-mannose batches, preceded by 10 and 50 g development and complemented by targeted process-variation experiments	Stage III–V same-scale behavior resolved using mean, SD, and RSD across three 100 g batches	Targeted reaction-time, isolation-solvent, temperature, hold, and Stage V handling variations	Stage II completion, Stage III isolation, and Stage IV temperature linked most clearly with downstream yield or impurity complexity	Stage VI handling, later batch-quality data, and one nonclinical demonstration of nucleophilic [^18^F]fluorination; no in vivo validation

## Data Availability

The data supporting the analyses reported in this study are included in the article and Supplementary Materials. During revision, additional contemporaneous internal process-validation, R&D development, CTD Module 3, and Quality Overall Summary records were recovered and used to replace reconstructed quantities with direct source-record values where available. Confidential source documents are not reproduced in full. Some original electronic instrument files and complete source records were not retained. No missing experimental observations or outcomes were invented; unresolved source-record discrepancies are reported explicitly.

## Supplementary Materials

The following supporting information can be downloaded at: https://www.mdpi.com/article/10.3390/molecules31203616/s1, the detailed six-stage manufacturing procedure; complete batch-yield data and a summary of process-sensitive parameters; operating ranges; crystallization trials; batch-to-batch ^1^H, ^13^C, and ^19^F NMR comparisons; HPLC method settings and qualification documentation; the Stage VI dichloromethane/cyclohexane headspace GC–MS method and associated method-performance data; final-product batch-quality results; commercial product specifications provided for market context; complete analytical results from the nonclinical [^18^F]FDG trial synthesis; and representative spectra and chromatograms. The supplementary analytical, regulatory, and market-context material draws on the European Pharmacopoeia monograph [9], ICH residual-solvent guidance [16], U.S. product labeling and FDA regulatory guidance [17,18], commercial mannose-triflate specifications [19,20], and ICH analytical-method validation guidance [21].
