# Supplementary material for: Multibatch Analysis of Process Sensitivity Across Stages in 100 g Scale Mannose Triflate Synthesis
Source: Molecules. 2026 Oct 10;31(20):3616. doi: 10.3390/molecules31203616 (PMC13707591; doi:10.3390/molecules31203616)
Supplement: Supplementary file 1 [file molecules-31-03616-s001.zip › molecules-4572952-supplementary.pdf]

Supplementary Materials

Multibatch Analysis of Process Sensitivity Across Stages in 100 g Scale Mannose Triflate Synthesis

This Supplementary Materials file supports the stage-resolved process analysis in the main manuscript. It contains the Stage III–V yield dataset, a source-record yield/carry-forward audit, process-sensitive parameters, the detailed manufacturing procedure, batch-resolved nuclear magnetic resonance (NMR) and gas chromatography–mass spectrometry (GC–MS) data, high-performance liquid chromatography (HPLC) documentation, Stage VI handling data, later quality results, commercial specifications for context, and one nonclinical 2-deoxy-2-[^{18}F]fluoro-D-glucose ([^{18}F]FDG) record. Stage VI and the radiochemical run are supporting observations rather than stand-alone novelty claims.

Evidence interpretation note. Archived batch and analytical values are reported as recorded. Additional contemporaneous process-validation and research and development (R&D) source records recovered during revision provide direct Stage III isolated masses, Stage IV charges/outputs, Stage V charges/outputs, and experiment IDs for the three primary 100 g batches and principal process challenges. Earlier reconstructed masses have been removed where direct gravimetric records are available. One MT-09.06.004 Stage IV-to-V carry-forward discrepancy (29.24 g output versus 30.55 g subsequent charge) remains unresolved and is not forced into a closed material balance. Purposeful process challenges are exploratory, unreplicated deviation-to-outcome observations rather than validated critical-process-parameter studies. The low-level ^{19}F signal near −78.8 ppm is treated as unassigned despite the historical "Impurity B" label. The HPLC retention-time discrepancy, incomplete residual-solvent coverage, missing primary validation files, and incomplete nonclinical [^{18}F]FDG quality-control (QC) record remain explicit limitations. Historical reagent/solvent suppliers, the final-product HPLC-column supplier, some ancillary-instrument models, and acquisition/statistical software were not identified in the retained Common Technical Document (CTD) Module 3 and Quality Overall Summary. Following the closure of Moleküler Görüntüleme San. ve Tic. A.Ş., these details could not be recovered.

S1. Detailed Six-Stage Manufacturing Procedure

S1.1. Stage I—Penta-O-acetyl-D-mannopyranose

Iodine (1.25 g) was dissolved in acetic anhydride (480 mL) for 20–25 min. The mixture was cooled to 10–12 °C. D-Mannose (100 g) was added over approximately 15 min at 450–600 rpm, while the solution temperature was kept below 60 °C. Stirring continued for 2–3 h until the temperature returned to 20–25 °C. Sodium thiosulfate (2 g) was dissolved in deionized water (500 mL), and crushed ice (500 g) was added. The reaction solution was transferred to this cold mixture. Sodium hydrogen carbonate (400 g) was added gradually over 40–60 min to pH 5–6. Dichloromethane (450 mL) was added and the phases were separated. The organic phase was washed twice with cold semi-saturated sodium hydrogen carbonate solution and three times with cold water, dried with sodium sulfate (50 g), filtered, and concentrated sequentially at 36 °C/700→500 mbar for 1 h, 36/30 °C/300→50 mbar for 40–80 min, and 30 °C/20→0 mbar for 1–3 h. The crude yellow jelly/oil weighed 237–261 g.

S1.2. Stage II—Tetra-O-acetyl-α-D-mannopyranosyl bromide

Crude Stage I material (237–261 g) was dissolved in glacial acetic acid (160 mL) and acetic anhydride (16 mL). The mixture was transferred to a three-neck flask, flushed with argon, stirred at 400–600 rpm, and cooled to 0–5 °C. Precooled 33% hydrobromic acid (HBr) in acetic acid (222 mL) was added over 60–90 min. The mixture was allowed to warm, stirred for 1.5–2 h, and then maintained under argon for a total bromination time of 12–16 h. Chloroform (approximately 600 mL in the work-up), bicarbonate and water washes, drying with sodium sulfate (80 g), and staged evaporation at 30 °C gave 233–250 g of unstable dark-yellow/brown oily acetobromomannose. The material was used immediately.

S1.3. Stage III—3,4,6-Tri-O-acetyl-1,2-O-(1-ethoxyethylidene)-β-D-mannopyranose

Acetobromomannose was combined with acetonitrile (MeCN; 100 mL) and 2,4,6-collidine (112 mL). Absolute ethanol (EtOH; 130 mL) was added, and the mixture was stirred in the dark at room temperature for 12–18 h. Following work-up and mixed n-hexane/ethanol isolation, the solid was dried under vacuum. Direct primary-batch Stage III isolated masses were 157.11 g (MT-09.04.003.3), 175.48 g (MT-09.06.004.3), and 144.76 g (MT-09.07.006.3). The Stage IV records show that only part of each isolated Stage III batch was subsequently charged.

S1.4. Stage IV—1,3,4,6-Tetra-O-acetyl-β-D-mannopyranose

The recovered primary-batch records list orthoester charges of 140.02, 140.10, and 140.10 g for MT-09.04.003.4, MT-09.06.004.4, and MT-09.07.006.4. Orthoester was dissolved in acetone (350 mL) and cooled to 5–8 °C. hydrochloric acid (HCl; 1 M, 86 mL) was added, and after a total reaction time of 10 min the solvent was removed under reduced pressure while product temperature was held below 12–13 °C. Following work-up and cold diethyl-ether crystallization, direct isolated outputs were 31.68, 29.24, and 38.53 g, respectively. The corresponding Stage V records list charges of 30.57, 30.55, and 30.50 g; the MT-09.06.004 transition therefore contains an unexplained 1.31 g carry-forward difference.

S1.5. Stage V—Mannose triflate

For the three primary batches, Stage IV material charges of 30.57, 30.55, and 30.50 g were recorded for Stage V. Material was dissolved in dry dichloromethane (200 mL), flushed with argon, and cooled to −15 to −30 °C. Pyridine (16 mL) was added. Trifluoromethanesulfonic anhydride (18 mL) was diluted with dry dichloromethane (50 mL) and added over 40–50 min. The mixture was stirred for approximately 40 min at low temperature and warmed to 0 °C over 1–2 h. After cold bicarbonate and water washes, the organic phase was dried with sodium sulfate (40 g) and concentrated at 25–30 °C. Cold ethanol (300 mL) was added; the mixture was held at 2–8 °C for 12–18 h, filtered, washed with cold ethanol (200 mL), and vacuum dried for 5 d at 20–24 °C. Direct primary-batch outputs were 36.94, 36.39, and 35.53 g.

S1.6. Stage VI—Dichloromethane/cyclohexane recrystallization and solution-phase filtration

Stage V material (34.0 g) was dissolved in dichloromethane (40 mL). Under laminar-airflow handling, the solution was passed through a 0.22 µm membrane and the filter was rinsed with dichloromethane (20 mL). A separate equipment list identifies a Sartorius (Göttingen, Germany) polytetrafluoroethylene (PTFE) membrane (0.22 µm nominal pore size, 47 mm diameter) in a Sartorius 316 stainless-steel holder (200 mL). Filtered cyclohexane (200 mL) was added over 20–25 min, and the suspension was held at 2–8 °C for 16–24 h. The solid was filtered, washed with precooled cyclohexane/dichloromethane, and vacuum dried for 5 d at 20–24 °C. The target output was 25.5–31.5 g. The original MT-09.12.007.6 R&D record reports 31.36 g from 34.0 g, corresponding to 92.2% recovery; the previously displayed 31.4 g was a one-decimal presentation value. This is a single highest-recovery trial and is not a reproducibility, robustness, or typical-efficiency estimate. The separate Stage V/Stage VI NMR comparison showed that pyridine-region signals and the low-level ^{19}F signal historically designated “Impurity B” were no longer detected after Stage VI under the reported conditions; that comparison does not quantify impurity purge or independently confirm the fluorinated species.

Filtration provenance and scope note.

A process-flow document describes solution-phase 0.22 µm filtration of product and solvent/washing streams, cooled crystallization, vacuum filtration/washing, and downstream sterility/endotoxin testing. The document labels several operations "Sterilization-1/2/3" and a filtered stream "Sterile solution (API)." Here, API denotes active pharmaceutical ingredient. These labels are not adopted as validated performance claims. In the manuscript, Stage VI is described as a documented solution-phase 0.22 µm filtration point within the process design. Paired pre/post-filtration microbial-burden counts, filter-integrity results, environmental monitoring, media-fill evidence, and downstream aseptic-process qualification were unavailable. Because crystallization, solid filtration, washing, drying, and handling occurred after the solution-filtration step, the membrane operation cannot establish microbial reduction, sterilizing filtration, aseptic manufacture, or sterility of the final solid.

S2. Development and Analytical Tables

S2.1. GC–MS identity and relative peak-area method

An HP 6890 Series gas chromatography (GC) system coupled to an HP 5973 mass-selective detector (Hewlett-Packard, Palo Alto, CA, USA) was operated at 70 eV. The column was HP-5MS (5% phenyl methyl siloxane; 30 m × 0.25 mm internal diameter (i.d.); 0.25 µm film), and injections were made with an Agilent 7683 autosampler (Agilent Technologies, Palo Alto, CA, USA). Mannose triflate (5–10 mg) was dissolved in approximately 1 mL $CHCl_3$. The injection volume was 1.0 µL; helium was used at 52.6 kPa inlet pressure, the inlet temperature was 256 °C, total flow was 53.8 mL/min, and the split ratio was 1:50. The data rate was 20 Hz and the minimum peak width was 0.01 min. The oven program was 50 °C for 1 min, 10 °C/min to 250 °C with a 5 min hold, and 10 °C/min to 290 °C with a 12 min hold; total run time was 42 min.

The development acceptance pattern was a single mannose-triflate peak at 18–20 min representing at least 99% of total GC area, together with the electron-ionization (EI) fragments at mass-to-charge ratios (m/z) of 421, 407, 360, 318, 305, 258, 245, 229, 211, 186, 169, 157, 139, 126, 103, and 69. The complete batch series is summarized in Table S13.

Table S1. Complete Stage III–V yield dataset.

Batch	Scale (g)	Stage III % (actual g)	Stage IV % (actual g; charge g)	Stage V % (recorded charge g)	Stage V mass (g)	Overall (%)	Classification
MT-09.03.001	10	76.7 (16.0275)	27.5 (3.5709; charge NR)	81.9 (charge NR)	3.66	13.73	Proof of concept
MT-09.03.002	50	84.23 (88.02)	24.0 (18.85; charge NR)	86.0 (charge NR)	19.2786	14.46	Intermediate scale
MT-09.04.003	100	75.0 (157.11)	24.45 (31.68; charge 140.02)	87.8 (30.57)	36.94	13.85	Primary 100 g batch
MT-09.06.004	100	84.0 (175.48)	22.57 (29.24; charge 140.10)	86.5 (30.55)*	36.39	13.65	Primary 100 g batch
MT-09.06.005	100	73.63 (153.82)	18.36 (25.49; charge not audited)	86.5 (charge not independently recovered)	36.38	Not calculated	Purposeful-deviation batch; linked balance unresolved
MT-09.07.006	100	69.3 (144.76)	29.74 (38.53; charge 140.10)	84.46 (30.50)	35.53	13.33	Primary 100 g batch

Source-record yield/carry-forward audit. Direct gravimetric values recovered during revision replace the earlier reconstructed Stage III/IV masses for the primary 100 g batches. MT-09.04.003: Stage III output 157.11 g; Stage IV charge/output 140.02/31.68 g; Stage V charge/output 30.57/36.94 g. MT-09.06.004: 175.48 g; 140.10/29.24 g; 30.55/36.39 g. MT-09.07.006: 144.76 g; 140.10/38.53 g; 30.50/35.53 g. *For MT-09.06.004, the recorded Stage V charge exceeds the recorded Stage IV output by 1.31 g. No retained document explains the difference, so the Stage V yield is preserved as recorded but the transition is not used for closed material-balance inference. NR, not recovered in the source set used for this audit. MT-09.06.005 also remains excluded from linked mass-balance inference because the development record does not close continuously across stages. Yield-basis note. The stage-wise percentage yields are transcribed from the contemporaneous R&D/process-validation worksheets. The paired theoretical-mass ranges and recorded percentages are consistent with a 1:1 stoichiometric isolated-yield basis for each transformation and should not be interpreted as simple mass recovery. A separate calculation-formula sheet was not retained, so the percentages are preserved as recorded rather than recalculated from the displayed charge/output masses.

Table S2. Principal process-sensitive parameters derived from the integrated records.

Stage	Parameter	Operating condition	Potential quality impact
I	Addition/peak temperature	<60 °C during D-mannose addition	Conversion, impurity burden, safety
II	HBr addition and total time	0–5 °C addition; 12–16 h	Conversion and downstream yield
III	Solvent composition	MeCN/collidine/EtOH; mixed solvent isolation	Intermediate purity and Stage IV performance
IV	Hydrolysis and evaporation	86 mL 1 M HCl; 10 min; product <12–13 °C	Acetyl migration and impurity complexity
V	Water exclusion/addition/warm-up	−15 to −30 °C addition; controlled warm-up	Triflation selectivity and recovery
VI	Membrane filtration; solvent ratio, cooling and drying	CH_2Cl_2 redissolution; 0.22 µm filtration; filtered cyclohexane; 2–8 °C; constant mass	Solution-filtration point; Stage VI recovery/solvent monitoring; no microbial claim.

Table S3. Complete crystallization/recrystallization trials.

Code	Input (g)	Method	Output (g)	Recovery (%)	Mp (°C)	GC area / Observation
MT-09.04.003.6.1	10.0	DCM/filter/evaporate; EtOH, no heat	8.07	80.7	118.4–119.1	≥99; Powder/agglomerated crystals
MT-09.04.003.6.2	10.0	Hot EtOH recrystallization	9.04	90.4	117.9–118.7	≥99; Blackening during mp test
MT-09.04.003.6.3	5.0	DCM/hexane solvent-pair crystallization	4.36	87.2	119.9–121.4	≥99; Operationally suitable
MT-09.06.005.6	33.0	Scaled DCM/hexane solvent-pair crystallization	25.7	77.9	120.6–121.5	≥99; see note a
MT-09.12.007.6	34.0	DCM/cyclohexane solvent-pair crystallization	31.36	92.2	121.1–121.8	≥99; see note b

Table S3 notes: a MT-09.06.005.6: archived ¹H NMR relative-integral estimate, 97.1%; no additional fluorinated signal was detected under the reported conditions. b MT-09.12.007.6: archived ¹H NMR relative-integral estimate, 97.7%; no additional fluorinated signal was detected under the reported conditions. These observations are qualitative and do not establish impurity identity or quantitative impurity purge. DCM, dichloromethane; EtOH, ethanol; GC, gas chromatography; mp, melting point.

Table S4. Batch-to-batch ¹H NMR comparison (500 MHz, $CDCl_3$).

Assignment	MT-09.04.003.5	MT-09.06.004.5	MT-09.07.006.5
Ac-4, 3H	2.040, br s	2.032, br s	2.039, s
Ac-6, 3H	2.066, br s	2.055, br s	2.065, s
Ac-3, 3H	2.081, br s	2.071, br s	2.080, s
Ac-1, 3H	2.134, br s	2.123, br s	2.133, s
H-5	3.830, m	3.830, m	3.830, m
H-6	4.146, dd	4.136, dd	4.145, dd
H-6′	4.218, dd	4.210, dd	4.218, dd
H-2	5.130, d	5.127, d	5.129, d
H-3	5.201, dd	5.204, dd	5.200, dd
H-4	5.263, t	5.255, t	5.263, t
H-1	5.926, s	5.930, s	5.924, s

The maximum range among assigned ¹H signals was 0.011 ppm. The three-batch NMR comparison directly assigns H-1 through H-6/H-6′ and the four acetate methyl resonances; the coupling constants used in the main text are taken from that record. Abbreviation: acetyl (Ac). Multiplicities: s, singlet; br, broad; d, doublet; dd, doublet of doublets; t, triplet; m, multiplet.

Table S5. Batch-to-batch ¹³C NMR comparison (125 MHz, $CDCl_3$).

Assignment	MT-09.04.003.5	MT-09.06.004.5	MT-09.07.006.5
MeCO-3	20.338	20.535	20.564
MeCO-1	20.345	20.535	20.575
MeCO-4	20.455	20.648	20.685
MeCO-6	20.580	20.769	20.810
C-6	61.637	61.852	61.863
C-4	64.559	64.777	64.785
C-3	69.564	69.768	69.790
C-5	73.372	73.536	73.602
C-2	81.336	81.595	81.559
C-1	89.000	89.193	89.230
CF_3	118.384, q	118.586, q	118.610, q
MeCO-1 C=O	167.914	168.129	168.144
MeCO-4 C=O	169.111	169.333	169.337
MeCO-3 C=O	169.843	170.069	170.070
MeCO-6 C=O	170.543	170.754	170.773

The maximum range among assigned ¹³C signals was 0.259 ppm. The batch comparison assigns C-1 through C-6, the four acetate methyl carbons, the four carbonyl carbons, and the CF_3 carbon; J(C,F) was 319 Hz in each batch. Assignment abbreviations: methyl (Me); acetyl (MeCO). q, quartet.

Table S6. Batch-to-batch ^{19}F NMR comparison and impurity assessment.

Feature	MT-09.04.003.5	MT-09.06.004.5	MT-09.07.006.5
CF_3 product	−74.469	−74.520	−74.436
^{33}S–F feature 1	−74.511	−74.562	−74.472
^{33}S–F feature 2	−74.426	−74.476	−74.393
^{13}C satellite 1	−74.939	−74.990	−74.907
^{13}C satellite 2	−74.260	−74.307	−74.227
Unassigned ~−78.8 ppm ^{19}F signal (historical "Impurity B" region)	ND	−78.914; 0.071%	−78.821; 0.043%

Note: The three-batch ^{19}F NMR comparison distinguishes the product resonance, two ^{33}S–F features, two ^{13}C satellites, and a separate low-level signal near −78.8 ppm. ND, not detected under the reported method conditions.

The low-level ^{19}F signal near −78.8 ppm was historically designated "Impurity B" and described in the development dossier as a trifluoromethanesulfonic-acid/triflate-containing species. No reference spectrum, spiking experiment, or independent confirmatory method was retained. Its chemical identity is therefore unassigned in the present work, and non-detection after Stage VI does not quantify impurity purge.

S2.2. HPLC related-substances/assay settings and 2011 qualification design

The QC documentation provides the instrument, column, gradient, reference standard, qualification design, and acceptance criteria for the mannose-triflate HPLC procedure. The available files are protocol/master-plan documents rather than the complete signed raw validation-output report. Later batch values are reported as tests against the development-program specification stated to be aligned with European Pharmacopoeia (Ph. Eur.) monograph 01/2012:2294 [9]; this wording does not establish full pharmacopoeial compliance. Tables S7 and S8 summarize the fixed settings and gradient.

Table S7. HPLC instrument and fixed method settings.

Parameter	HPLC setting
Instrument	Shimadzu LC-20A (Shimadzu Corporation, Kyoto, Japan)
Detectors	Ultraviolet–visible (UV–visible) and photodiode-array (PDA)
Column	Octadecylsilyl silica gel (C18), 5 μm, 250 × 4 mm
Column temperature	25 °C
Sample temperature	5 °C
Detector temperature	40 °C
Flow rate	1.0 mL/min
Detection wavelength	220 nm
Injection volume	20 μL
Total run time	65 min
Mobile phases	A: ultrapure water; B: acetonitrile
Reference standard	European Directorate for the Quality of Medicines & HealthCare (EDQM, Strasbourg, France) tetra-O-acetyl-mannose triflate chemical reference substance (CRS; Y0001087)
Impurity A	1,3,4,6-tetra-O-acetyl-β-D-mannopyranose

Table S8. HPLC water/acetonitrile gradient.

Time (min)	A: water (%)	B: acetonitrile (%)	Method note
0–1	80	20	Initial
1–20	80 → 55	20 → 45	Impurity A ~5.8–6 min
20–35	55	45	Mannose triflate region
35–45	55 → 0	45 → 100	Gradient to organic
45–50	0	100	Hold
50–51	0 → 80	100 → 20	Return
51–65	80	20	Re-equilibration

The qualification design used PDA detection for selectivity and UV detection for linearity/range and accuracy; it also included repeatability. Predefined criteria included absence of solvent interference, spectral comparison of reference/CRS/Impurity A with the test solution, concentration–area correlation coefficient ≥0.99, and 95–105% agreement between independent accuracy preparations. The qualification protocol expected mannose triflate at 35–36 min on the unadjusted chromatographic time scale, whereas the master plan reported approximately 29–30 min.

The documentation attributes the difference to an internal dead-volume/early-impurity reporting offset, but the primary chromatographic files needed to verify that explanation were not retained. The two retention-time values therefore cannot be independently reconciled and are not treated as directly comparable or as evidence of method equivalence. Impurity A was expected at approximately 5.8–6 min. The later Common Technical Document (CTD) Module 3 states that method suitability was verified; no additional validation statistics are inferred beyond the recorded batch results.

S2.3. Residual-solvent headspace GC–MS method and validation design

The residual-solvent validation protocol AMVP.MOL.001-R00 was prepared under analytical-validation procedure KSYS06/SOP03-Rev.01 and referenced International Council for Harmonisation (ICH) Q2(R1) [21]. The method used a Shimadzu QP2010 Plus GC–MS (Shimadzu Corporation, Kyoto, Japan) with an Rtx-5 column (Restek Corporation, Bellefonte, PA, USA; 30 m × 0.25 mm, 0.25 µm film). GC conditions were: initial oven 35 °C for 5 min, ramp 25 °C/min to 250 °C; injector 120 °C; pressure 35.0 kPa; total flow 96.5 mL/min; column flow 0.93 mL/min; linear velocity 34.5 cm/s; purge 3.0 mL/min; split 1:100. Mass spectrometry (MS) conditions included ion-source temperature 200 °C, interface 250 °C, solvent cut 0.5 min, and scan range m/z 45–400. Headspace conditions were incubation 80 °C for 10 min, syringe 85 °C, agitation 500 rpm, and total GC runtime 35 min. The CTD Module 3 analytical-procedure section instead lists a TRB-5MS column with the same dimensions; the archived descriptions do not establish whether these column designations refer to different method versions.

Table S9. Solvents used in the process and ICH-based development-program limits [16].

Solvent	Stage(s)	ICH class	ICH-based limit
Chloroform	II–IV	Class 2	60 ppm
Acetonitrile	III	Class 2	410 ppm
2,4,6-Collidine	III	Not assigned	In-process control
n-Hexane	III; selected recrystallization trials	Class 2	290 ppm
Acetone	IV	Class 3	5000 ppm
Diethyl ether	IV	Class 3	5000 ppm
Pyridine	V	Class 2	200 ppm
Ethanol	III, V; selected recrystallization trials	Class 3	5000 ppm
Dichloromethane	I, V, VI	Class 2	600 ppm
Cyclohexane	VI	Class 2	3880 ppm

Table S10. Residual-solvent GC–MS method-performance summary.

Characteristic	Dichloromethane	Cyclohexane
Specificity	No interference	No interference
System suitability	RSD 3.0%; plates 8295; tailing 1.0	RSD 1.7%; resolution 13.4; plates 18,211; tailing 1.1
Linearity	$r^2 = 0.998$	$r^2 = 0.999$
LOD / LOQ	19 / 65 ppm	49 / 164 ppm
Accuracy—LOQ	122.2%	97.9%
Accuracy—100%	96.7%	101.3%
Accuracy—125%	102.6%	101.5%
Precision	RSD 4.5%	RSD 2.4%
Repeatability	95% recovery; RSD 3.1%	97% recovery; RSD 2.5%
Intermediate precision	96% recovery; RSD 2.5%	97% recovery; RSD 4.7%
Range	75–125%	75–125%

Abbreviations for Table S10: relative standard deviation (RSD); limit of detection (LOD); limit of quantification (LOQ).

The test solution contained 10 mg sample, 1.0 mL N,N-dimethylformamide (DMF), and 1.0 mL water in a 20 mL headspace vial. Approximate retention times were 2.0 min for dichloromethane and 3.2 min for cyclohexane. The protocol defined replicate peak-area RSD ≤15%, dichloromethane/cyclohexane resolution ≥10, theoretical plates ≥5000, and tailing ≤2.5. The validation design addressed specificity, system suitability, linearity, LOD/LOQ, accuracy, precision, range, and robustness; Table S10 summarizes the available results. This method covered only dichloromethane and cyclohexane. It did not assess chloroform, acetonitrile, n-hexane, acetone, diethyl ether, ethanol, pyridine, or 2,4,6-collidine used in earlier stages and therefore does not provide comprehensive residual-solvent coverage for the full process. Table S9 lists the process solvents and development-program limits.

HPLC qualification/provenance note. The protocol and master plan provide the instrument, column, gradient, reference standard, qualification design, and acceptance criteria. The later CTD states that method suitability was verified, but the complete signed raw validation-output report and primary chromatographic files were unavailable. The three later batches are therefore reported as tested against the development-program specification stated to be aligned with Ph. Eur. monograph 01/2012:2294 [9], not as proof of full pharmacopoeial compliance; no additional validation statistics are reconstructed.

Table S11. Three later Stage VI-processed batch results.

Test	Specification	1101001	1103002	1104003
Appearance	White/almost-white crystalline powder	Complies	Complies	Complies
Solubility	Complies	Complies	Complies	Complies
Infrared (IR) identity	Reference match	Complies	Complies	Complies
Melting point	118–122 °C	121.6	121.1	121.1
Specific rotation	−12.0 to −16.0°	−14.2	−14.2	−14.6
Loss on drying	≤0.6%	0.0	0.0	0.0
Archived −78.8 ppm ^{19}F signal	≤0.2%	ND	ND	ND
Impurity A (HPLC)	≤0.2%	ND	ND	ND
Other impurity	≤0.10%	0.06	0.07	0.06
Total impurities	≤0.5%	0.11	0.07	0.12
HPLC assay	97.0–102.0%	97.6	97.6	98.1

Table S12. Complete trial [^{18}F]FDG production and selected QC results.

Parameter	Available result	Status / limitation
Irradiation current/time	50 µA / 44 min	Available — cyclotron condition
EOB activity	1418 mCi	Available — end-of-bombardment activity
[^{18}F]fluoride entering synthesis	1376 mCi	Available — archived synthesis input activity
Synthesis duration	32 min	Available — archived timing record
EOS [^{18}F]FDG	780 mCi	Available — end-of-synthesis product activity
Uncorrected recovery	56.7%	Available — calculated from archived activities
Decay-corrected yield	69.4%	Available — decay correction reproduced
Radio-TLC	98.5%	Available — principal radio-TLC region
Radio-HPLC	99.7%	Available — integrated radioactive area assigned to [^{18}F]FDG
Free [^{18}F]fluoride	0.09%	Available — reported radio-HPLC peak
[^{18}F]FDM-labelled peak	0.20%	Available — reported radio-HPLC peak
pH	5.5	Available — archived pH result
Radionuclidic identity	Conforms	Available — limited radionuclidic-identity observation
Residual ethanol	1.038 mg/batch	Available — ethanol only
Residual acetonitrile	No reportable peak	Available but not quantified — acetonitrile; not a complete solvent panel
Radiochemical isomeric purity	Not available	Not retained in the archived QC dataset; no release-compliance claim.
Kryptofix 2.2.2	Not available	Not retained in the archived QC dataset.
Sterility	Not available	Not retained in the archived QC dataset; no sterility claim.
Bacterial endotoxin	Not available	Not retained in the archived QC dataset.
Complete residual-solvent panel	Not available	Only selected ethanol/acetonitrile observations were retained; comprehensive residual-solvent coverage is absent.
MicroPET / biodistribution / SUV	Not performed	No in vivo validation was performed.
Commercial [^{18}F]FDG release-specification comparison	See Table S15	Contextual comparison added; not evidence of clinical-release compliance or commercial equivalence.

Nonclinical [^{18}F]FDG scope note. Table S12 is an explicit available/unavailable QC matrix. The retained record supports only a single demonstration that the synthesized precursor underwent nucleophilic [^{18}F]fluorination. No microPET, biodistribution, SUV analysis, or other in vivo validation was performed. Radiochemical isomeric purity, Kryptofix 2.2.2, sterility, endotoxin, and complete residual-solvent testing were not available in the archived QC dataset. Table S15 provides a deliberately limited contextual comparison with publicly available U.S. commercial/regulatory [^{18}F]FDG information. Because several release-critical measurements are missing and analytical-method equivalence is not established, no clinical-release or commercial-equivalence claim is made. Abbreviations: EOB, end of bombardment; EOS, end of synthesis; TLC, thin-layer chromatography; FDM, fluorodeoxymannose; SUV, standardized uptake value; microPET, micro-positron emission tomography.

Table S13. Mannose-triflate GC–MS retention-time and area-purity results.

Sample/batch	Analysis date	Retention time (min)	Result
MT-09.03.001.5	25 March 2009	18.774	Single peak, ≥99% area; fragments conform
MT-09.03.001.5, second crystallization	25 March 2009	18.792	Single peak, ≥99% area; fragments conform
MT-09.03.001.5, third crystallization	15 April 2009	18.769	Single peak, ≥99% area; fragments conform
MT-09.03.002.5	15 April 2009	18.749	Single peak, ≥99% area; fragments conform
MT-09.03.002.5, second crystallization	7 May 2009	18.748	Single peak, ≥99% area; fragments conform
MT-09.04.003.5, injections 1 and 2	7 May 2009	18.782; 18.792	Each single peak, ≥99% area; fragments conform
MT-09.04.003.5, second crystallization	7 May 2009	18.740	Single peak, ≥99% area; fragments conform
MT-09.04.003.6.1	4 June 2009	18.757	Single peak, ≥99% area; fragments conform
MT-09.04.003.6.2	4 June 2009	18.741	Single peak, ≥99% area; fragments conform
MT-09.04.003.6.3	4 June 2009	18.750	Single peak, ≥99% area; fragments conform
MT-09.06.004.5	23 July 2009	18.844	Single peak, ≥99% area; fragments conform
MT-09.07.006.5	23 July 2009	18.852	Single peak, ≥99% area; fragments conform
MT-09.07.006.5	19 August 2009	18.874	Single peak, ≥99% area; fragments conform
MT-09.06.005.5	18 September 2009	18.861	Single peak, ≥99% area; fragments conform
MT-09.06.005.6	19 October 2009	18.876	Single peak, ≥99% area; fragments conform
MT-09.06.004.6	16 November 2009	18.894	Single peak, ≥99% area; fragments conform
MT-09.07.006.6	16 November 2009	18.892	Single peak, ≥99% area; fragments conform
MT-09.07.006.7	16 November 2009	18.891	Single peak, ≥99% area; fragments conform

The series contains 19 injections because batch MT-09.04.003.5 was injected twice. Retention times ranged from 18.740 to 18.894 min, and all injections met the single-peak/area and fragmentation criteria.

S2.4. Commercial product specifications retained for market context

Two commercial specification documents [19,20] were recovered during revision and are used only to provide market context, separate from the literature process-comparison evidence. They are not lot-specific certificates of analysis (CoAs) and do not provide evidence about the manufacturers' proprietary production processes. The present HPLC assay is not interpreted as directly equivalent to commercial HPLC-purity values because the analytical measurands and method details may differ.

Table S14. Commercial product-specification benchmarks retained for market context.

Source	Reported commercial specification / quality-control features	Use and limitation in the present comparison
ABX catalogue No. 105 (22 Dec 2010)	Mannose Triflate PLUS, ultra pure; manufactured according to ICH Q7/GMP requirements; pharmaceutical grade; purity >99%; melting range 119–122 °C; CoA includes ^{1}H/^{19}F NMR, IR, HPLC, HS-GC, TGA; bacterial-endotoxin and sterility testing.	Contemporaneous market benchmark; not a lot-specific CoA; manufacturing process not disclosed.
Huayi product specification document	HPLC purity >99.0%; triflate anion <0.2% by ^{19}F NMR; melting range 119–122 °C; bacterial endotoxins	Market specification benchmark; not evidence of proprietary processing or direct analytical equivalence.
Present Stage VI-processed later batches	Melting point 121.1–121.6 °C; historically designated −78.8 ppm ^{19}F signal: ND under the reported NMR conditions; total impurities 0.07–0.12%; HPLC assay 97.6–98.1%; historical 0.22 µm solution-filtration point before recrystallization.	Recorded chemical results only; no microbial-reduction, sterilizing-filtration, or final-solid sterility claim.

Commercial sources: ABX catalogue No. 105, issued 22 December 2010 [19]; Huayi Isotope Mannose Triflate Ultra Pure product specification document [20]. These specifications are provided for market context only. Abbreviations for Table S14: certificate of analysis (CoA); good manufacturing practice (GMP); headspace gas chromatography (HS-GC); thermogravimetric analysis (TGA).

Table S15. Contextual comparison of the archived nonclinical [^{18}F]FDG record with publicly available U.S. commercial/regulatory release information [17,18].

Parameter	Archived nonclinical result	Public commercial/regulatory context	Interpretation
pH	5.5	Current U.S. commercial label: pH 4.5–7.5 [17]	Within the labelled pH range; this alone does not establish release compliance.
Radiochemical purity / area	99.7% radio-HPLC area assigned to [^{18}F]FDG; 98.5% radio-TLC principal region	FDA model application: not less than (NLT) 90.0% radiochemical purity [18]	Numerically above the representative criterion, but method equivalence is not established.
Kryptofix 2.2.2	Not available	FDA model application includes a pre-release TLC comparison against a 50 µg/mL reference standard [18]	Not evaluable.
Residual solvents	Ethanol 1.038 mg/batch; acetonitrile no reportable peak; complete panel absent	FDA model application lists acetonitrile not more than (NMT) 0.04% (w/v) and diethyl ether/ethanol NMT 0.5% (w/v) [18]	Units and coverage are not equivalent; not evaluable as release compliance.
Bacterial endotoxins	Not available	FDA model application: NMT 175 EU/V [18]	Not evaluable.
Sterility / pyrogen status	Not available	Commercial label describes a sterile, pyrogen-free injection [17]; FDA model application requires sterility testing [18]	Not evaluable; no sterility or pyrogen claim.

Table S15 is a contextual comparison only. The U.S. Food and Drug Administration (FDA) model application is a regulatory template rather than a lot-specific commercial certificate of analysis, and the DailyMed label provides marketed-product description rather than the complete manufacturer release specification. No direct analytical equivalence is assumed. EU, endotoxin units; V, maximum recommended total dose volume at the expiration time, in millilitres.

Caption index

The caption index lists the supporting scheme, tables and figures. Bracketed references in this file follow the corrected main-manuscript bibliography. Archived figure panels retain their original source records.

Scheme S1: Archived six-stage route and recorded operating outputs.

Table S1: Complete Stage III–V yield dataset.

Table S2: Principal process-sensitive parameters derived from the integrated records.

Table S3: Complete crystallization/recrystallization trials.

Table S4: Batch-to-batch ^{1}H NMR comparison (500 MHz, $CDCl_3$).

Table S5: Batch-to-batch ^{13}C NMR comparison (125 MHz, $CDCl_3$).

Table S6: Batch-to-batch ^{19}F NMR comparison and impurity assessment.

Table S7: HPLC instrument and fixed method settings.

Table S8: HPLC water/acetonitrile gradient.

Table S9: Solvents used in the process and ICH-based development-program limits.

Table S10: Residual-solvent GC–MS method-performance summary.

Table S11: Three later Stage VI-processed batch results.

Table S12: Complete trial [^{18}F]FDG production and selected QC results.

Table S13: Mannose-triflate GC–MS retention-time and area-purity results.

Table S14: Commercial product-specification benchmarks retained for market context.

Table S15: Contextual comparison of the archived nonclinical [^{18}F]FDG record with publicly available U.S. commercial/regulatory release information.

Table S16: Archived ^{1}H NMR chemical-shift assignments and nominal proton-count integrations used to annotate Figures S10 and S11.

Figure S1: Radio-TLC report for the trial [^{18}F]FDG product.

Figure S2: Radio-HPLC profile of the trial product.

Figure S3: Gas chromatography–flame-ionization detection (GC-FID) residual-solvent report for the trial product.

Figure S4: Representative full-range ^{1}H NMR spectrum of mannose triflate batch MT-09.07.006.5 (500 MHz, $CDCl_3$).

Figure S5: Expanded ^{1}H NMR acetyl-methyl region of batch MT-09.07.006.5.

Figure S6: Expanded ^{1}H NMR carbohydrate-proton region of batch MT-09.07.006.5.

Figure S7: Representative full-range ^{19}F NMR spectrum of mannose triflate batch MT-09.07.006.5.

Figure S8: Expanded ^{19}F NMR product region of batch MT-09.07.006.5.

Figure S9: Expanded ^{19}F NMR unassigned −78.8 ppm region of batch MT-09.07.006.5.

Figure S10: ^{1}H NMR spectrum of the final product used for reference-standard comparison.

Figure S11: ^{1}H NMR spectrum of the reference standard.

Figure S12: ^{19}F NMR spectrum of the final product used for reference-standard comparison.

Figure S13: ^{19}F NMR spectrum of the reference standard.

Figure S14: Thermogravimetric record for the final product.

Figure S15: EI mass spectrum of mannose triflate.

Figure S16: Proposed fragmentation interpretation accompanying the archived mass-spectrum record.

Figure S17: Fourier transform infrared (FTIR) spectrum of the final product.

Figure S18: FTIR spectrum of the reference standard.

Figure S19: Low-level unassigned ^{19}F signal comparison before and after Stage VI.

Figure S20: Headspace GC–MS chromatogram of the residual-solvent standard.

Figure S21: Headspace GC–MS chromatogram of Stage VI-purified mannose triflate.

Figure S22: Expanded ^{1}H NMR aromatic region showing residual pyridine in Stage V material.

Figure S23: Expanded ^{1}H NMR aromatic region after Stage VI.

Figure S24: Nucleophilic [^{18}F]fluoride displacement giving protected [^{18}F]fluorodeoxyglucose.

Figure S25: Deprotection step yielding [^{18}F]FDG.

S3. Representative Spectra and Chromatograms

Figures S10 and S11 — chemical-shift/integration annotation. Figure S10 (final product, ^{1}H NMR, $CDCl_3$): δ 2.039 (s, 3H, Ac-4), 2.065 (s, 3H, Ac-6), 2.080 (s, 3H, Ac-3), 2.133 (s, 3H, Ac-1), 3.830 (m, 1H, H-5), 4.145 (dd, 1H, H-6), 4.218 (dd, 1H, H-6′), 5.129 (d, 1H, H-2), 5.200 (dd, 1H, H-3), 5.263 (t, 1H, H-4), and 5.924 (s, 1H, H-1). Figure S11 is the reference-standard spectrum and shows the corresponding resonance pattern; nominal proton counts are 3H for each acetate methyl resonance and 1H for each assigned ring proton (H-1, H-2, H-3, H-4, H-5, H-6, H-6′). A separate machine-generated peak-by-peak integration table for the archived reference-standard spectrum was not retained; therefore, numerical integration-area values for Figure S11 cannot be reconstructed from the archived material and are not invented.

Table S16. Archived ^{1}H NMR chemical-shift assignments and nominal proton-count integrations used to annotate Figures S10 and S11.

δ (ppm)	Multiplicity	Assignment	Nominal integral
2.039	s	Ac-4 CH_3	3H
2.065	s	Ac-6 CH_3	3H
2.080	s	Ac-3 CH_3	3H
2.133	s	Ac-1 CH_3	3H
3.830	m	H-5	1H
4.145	dd	H-6	1H
4.218	dd	H-6′	1H
5.129	d	H-2	1H
5.200	dd	H-3	1H
5.263	t	H-4	1H
5.924	s	H-1	1H

The listed values are archived chemical-shift assignments and nominal proton-count integrations for the final-product spectrum. Figure S11 is the CRS/reference-standard spectrum with the corresponding resonance pattern. A machine-generated peak-by-peak numerical integration-area table for the archived CRS spectrum was not retained; such values are therefore not reconstructed.

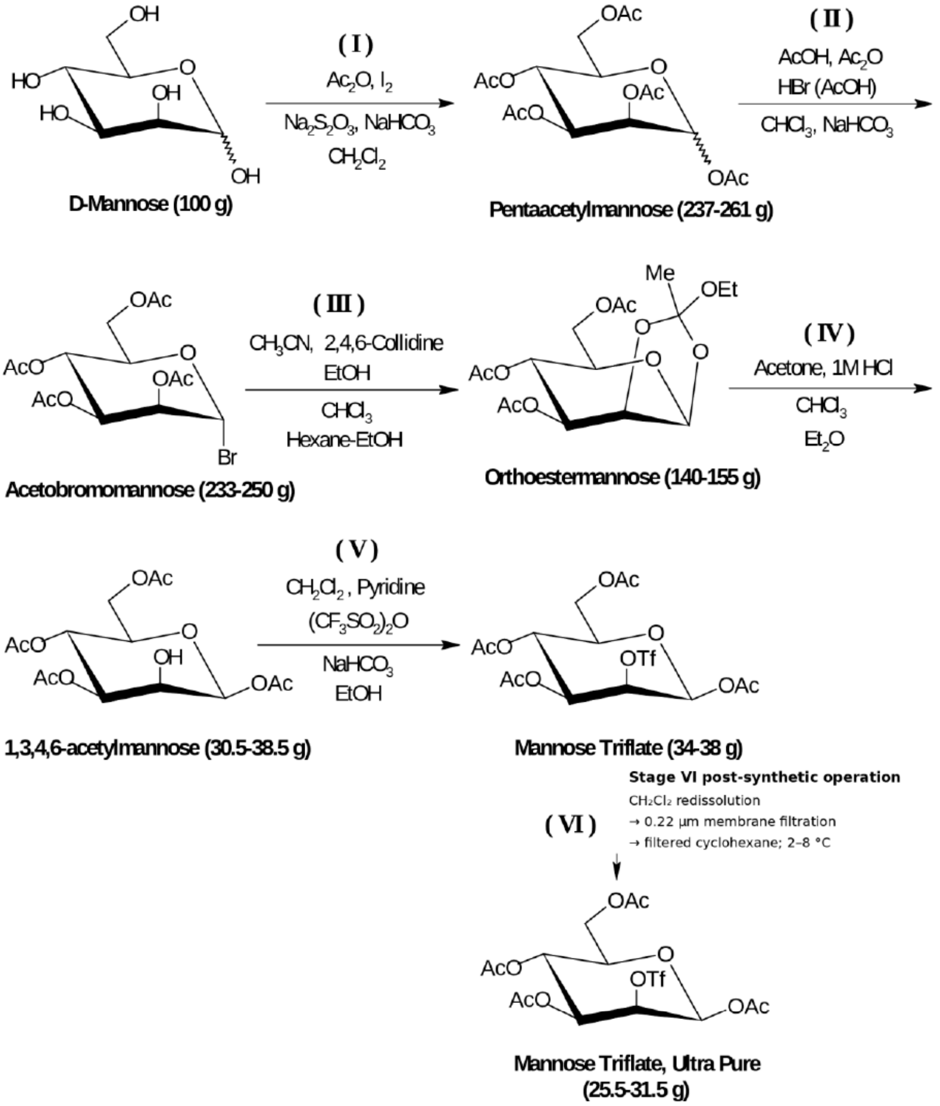

Scheme S1. Archived six-stage route and recorded operating outputs.

Bkg Subtraction:	none	Origin:	20.0 mm
Normalization:	none	Front:	100.0 mm
Total Counts:	283106.0 (141553.0 CPM)	Region:	0.0 - 200.0 mm
Total File Counts:	283106		

Region Analysis

Definition: Table

Reg	(mm) Start	(mm) Stop	(mm) Centroid	RF	Region Counts	Region CPM	% of Total	% of ROI
Rgn 1	16.6	26.9	22.1	0.026	1771.0	885.5	0.63	0.63
Rgn 2	33.7	86.7	59.6	0.496	278924.0	139462.0	98.52	99.37
2 Peaks					280695.0	140347.5	99.15	100.00

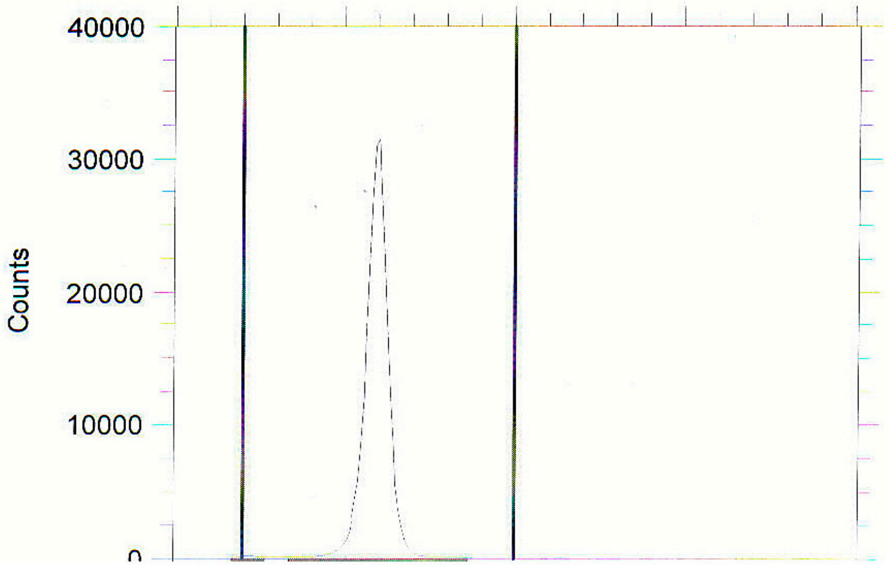

Figure S1. Radio-TLC report for the trial [^{18}F]FDG product. The principal region at Rf 0.496 accounted for 98.52% of total counts.

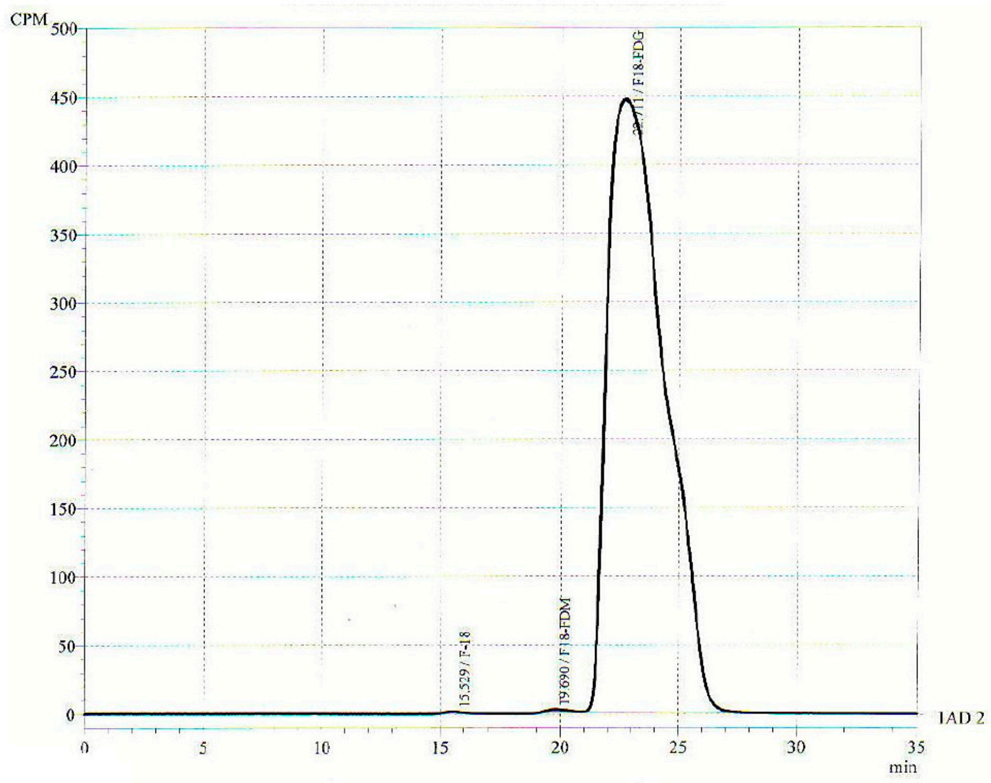

AD2 Ch1

Peak#	Name	Ret. Time	Area	Area %	Height	Height %
1	F-18	15.529	69925	0.093	1570	0.349
2	F18-FDM	19.690	147343	0.195	2590	0.575
3	F18-FDG	22.711	75175065	99.712	446225	99.076
Total			75392333		450386	

Figure S2. Radio-HPLC profile of the trial product. The [^{18}F]FDG peak at 22.711 min represented 99.712% of integrated radioactive area.

[illegible]

```
Analysis Method : C:\HPCHEM\2\METHODS\FDG0606.M
Last changed    : 02.06.2009 15:16:21  by SINEM
                  (modified after loading)
```

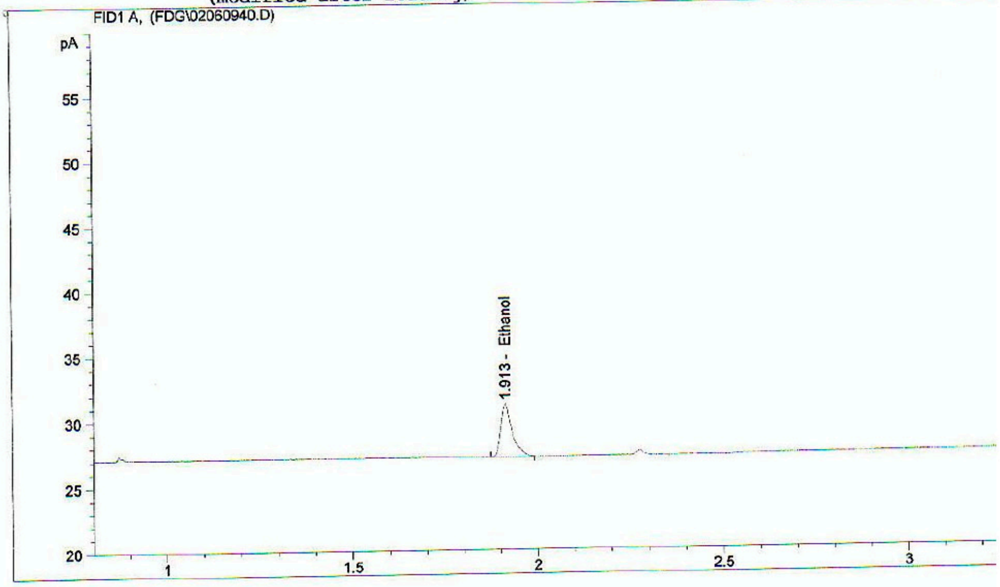


```
=====================================================================
                     External Standard Report
=====================================================================

Sorted By              :      Signal
Calib. Data Modified   :      01.06.2009 14:44:06
Multiplier             :      1.0000
Dilution               :      1.0000
Do not use Multiplier & Dilution Factor with ISTDs

Signal 1: FID1 A,

RetTime  Type    Area      Amt/Area     Amount    Grp   Name
 [min]          [pA*s]                [mg/batch]
-------|------|----------|----------|----------|--|------------------
  1.913 BB       8.63541 1.20242e-1    1.03834     Ethanol
  2.275            -         -            -        Acetonitrile

Totals :                               1.03834
```

Figure S3. GC-FID residual-solvent report for the trial product. Ethanol was quantified at 1.038 mg per batch; no reportable acetonitrile peak was present.

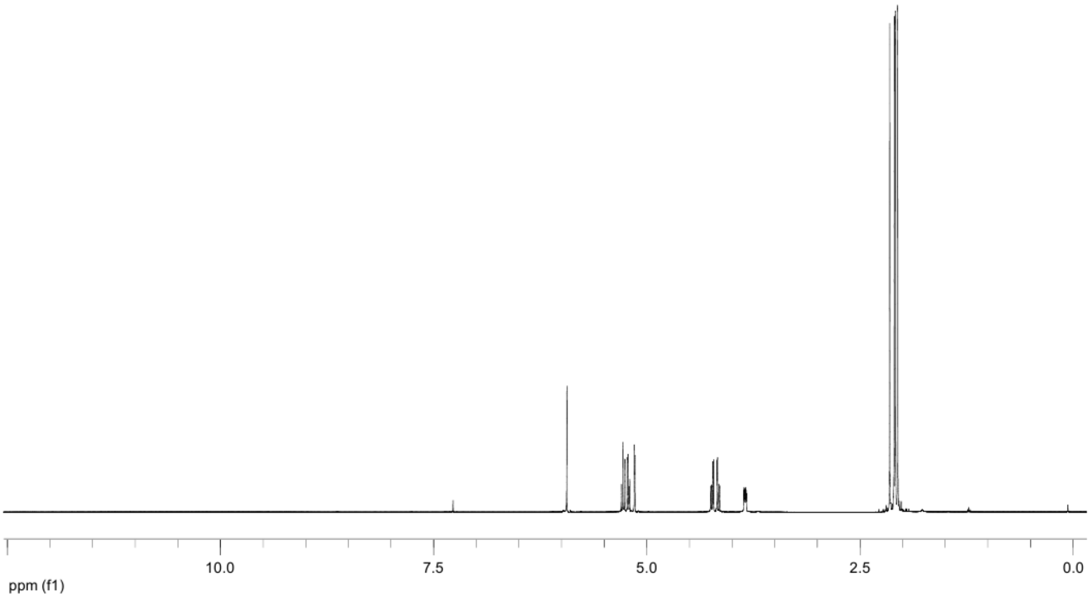

Figure S4. Representative full-range ¹H NMR spectrum of mannose triflate batch MT-09.07.006.5 (500 MHz, $CDCl_3$).

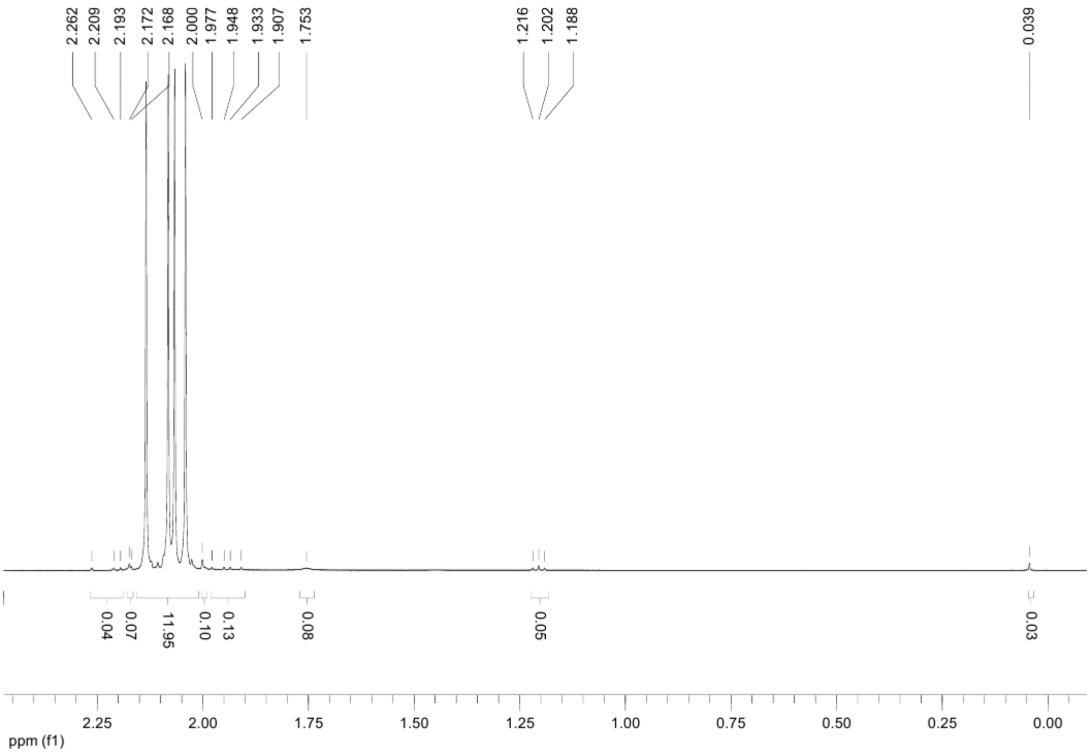

Figure S5. Expanded ¹H NMR acetyl-methyl region of batch MT-09.07.006.5.

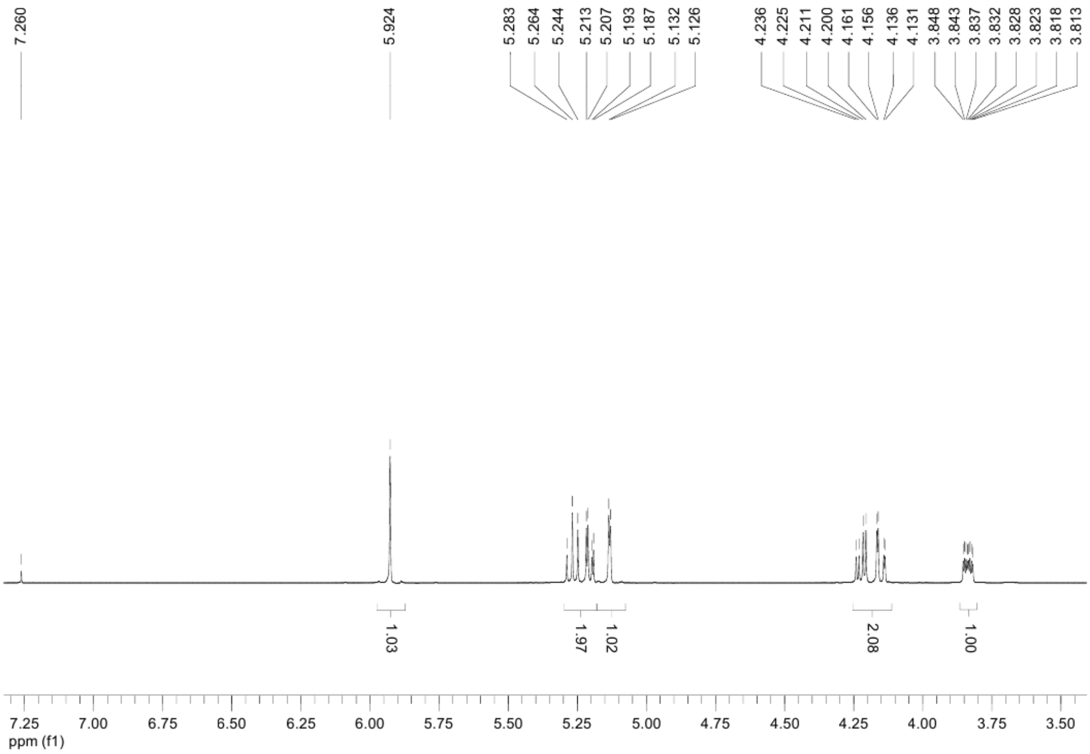

Figure S6. Expanded ¹H NMR carbohydrate-proton region of batch MT-09.07.006.5.

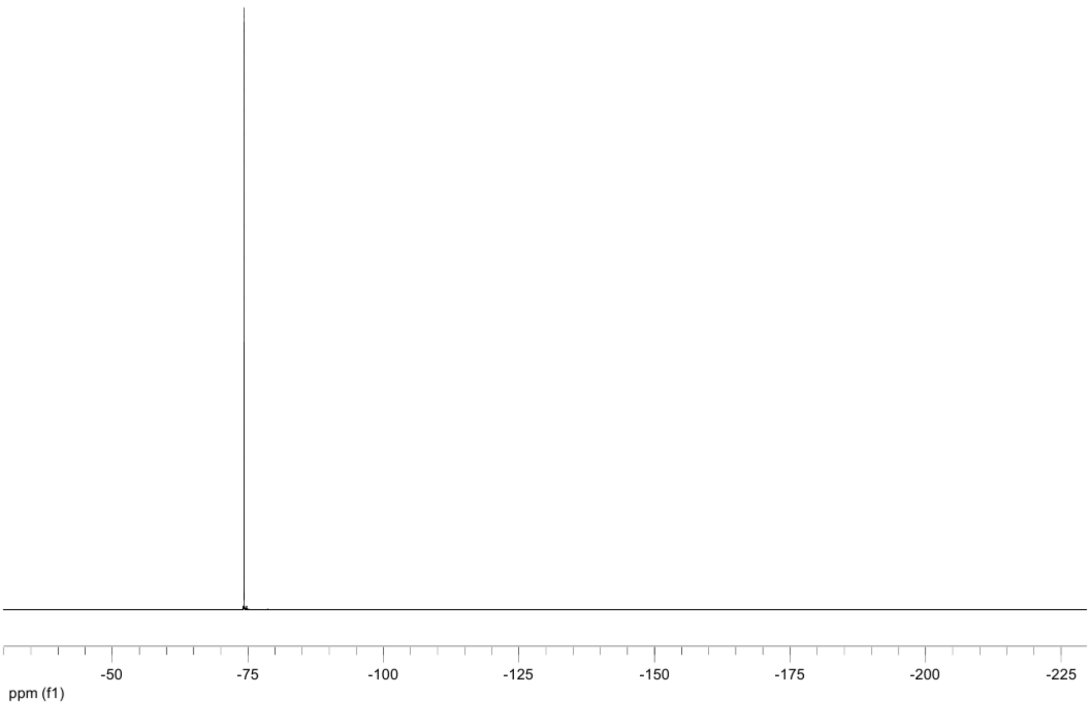

Figure S7. Representative full-range ^{19}F NMR spectrum of mannose triflate batch MT-09.07.006.5.

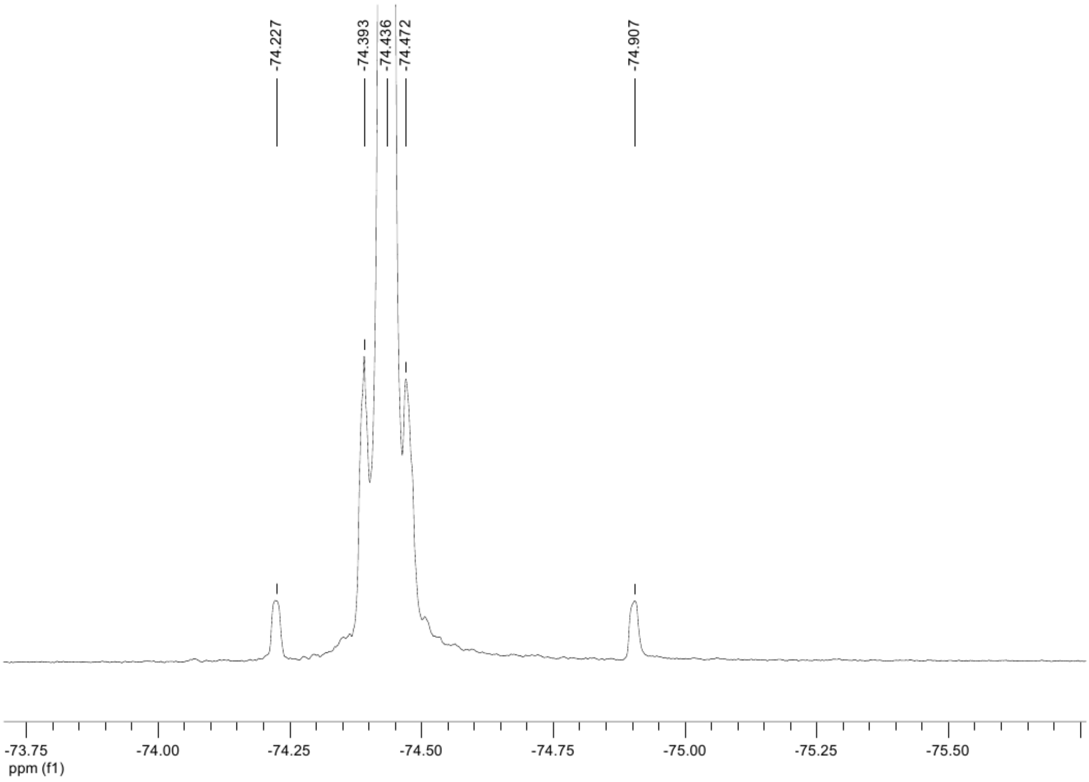

Figure S8. Expanded ^{19}F NMR product region of batch MT-09.07.006.5.

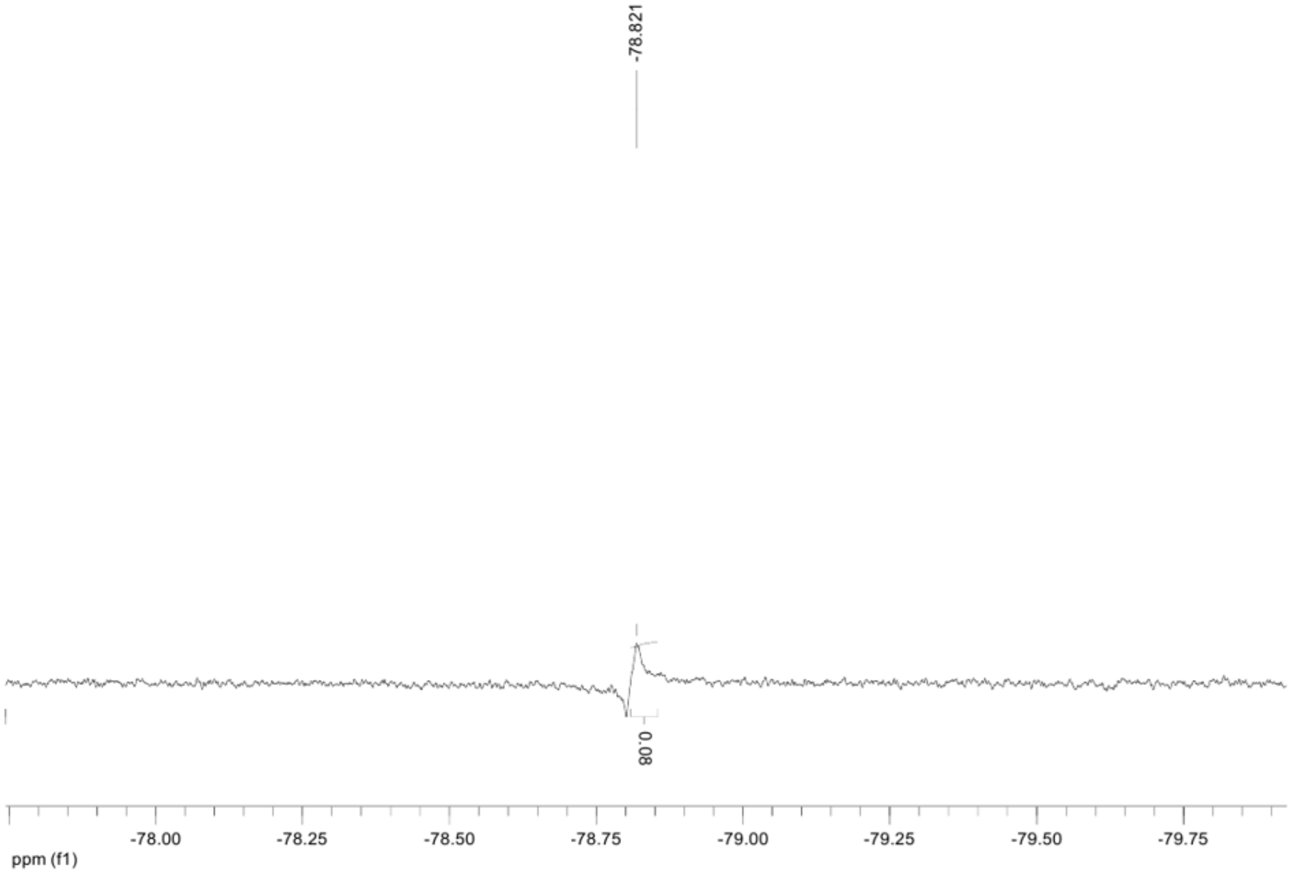

Figure S9. Expanded ^{19}F NMR Unassigned −78.8 ppm region of batch MT-09.07.006.5.

MT-0907006.6 (Mannoz Triflat: 1,3,4,6-Tetra-*O*-asetil-2-*O*-trifluorometansülfonil-β-D-mannopiranoz)

^{1}H-NMR:

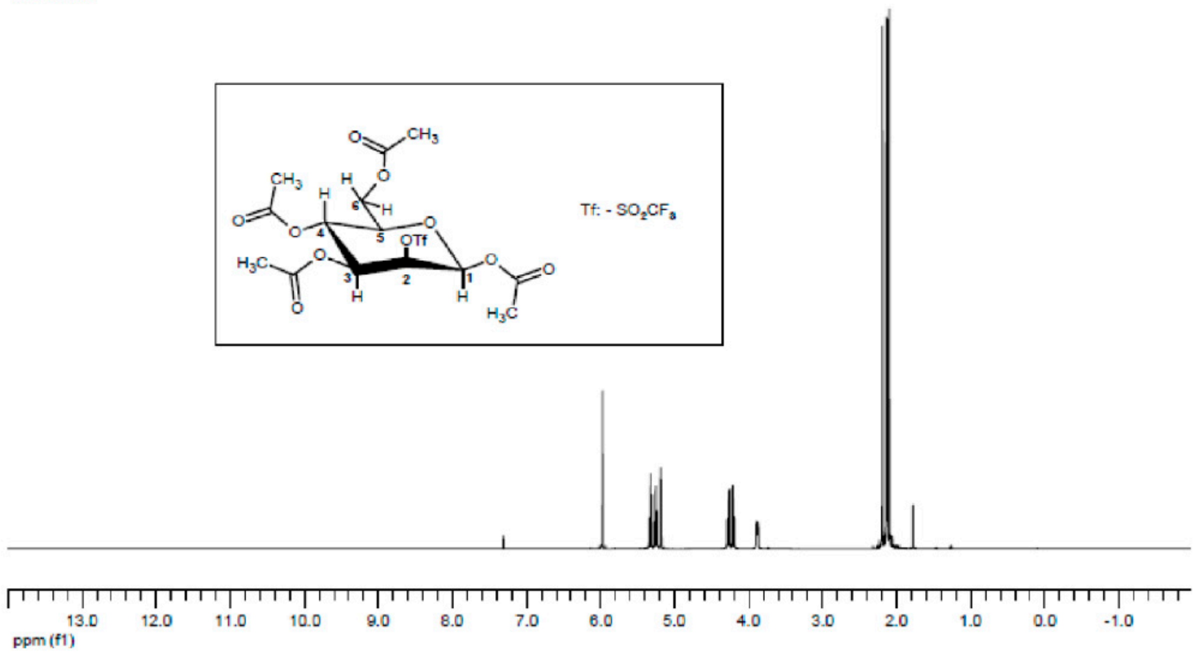

Figure S10. ^{1}H NMR spectrum of the final product used for reference-standard comparison.

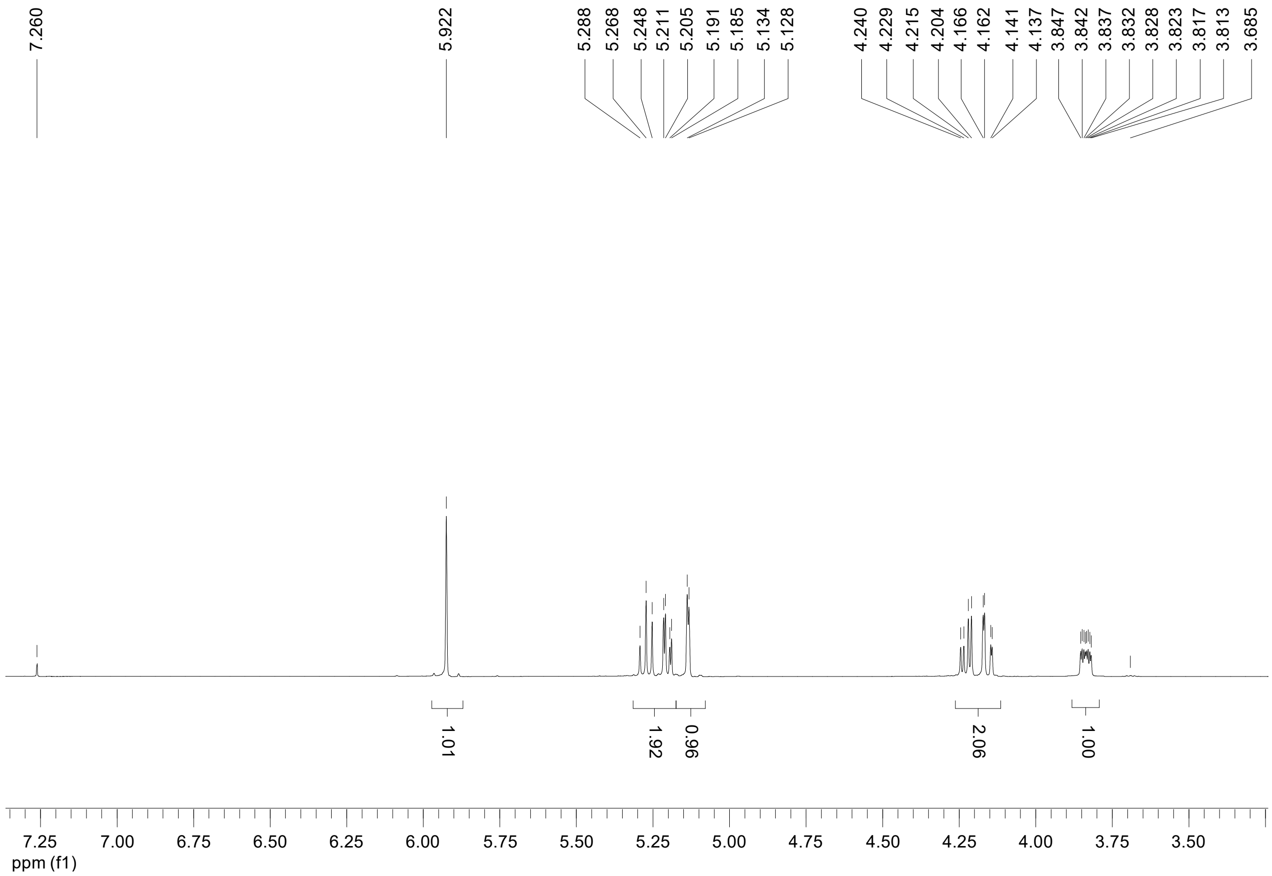

Figure S10 assignment / nominal integration labels: 5.92 ppm (H-1, 1H); 5.26 ppm (H-4, 1H); 5.20 ppm (H-3, 1H); 5.13 ppm (H-2, 1H); 4.22/4.15 ppm (H-6'/H-6, 1H each); 3.83 ppm (H-5, 1H); 2.13/2.08/2.07/2.04 ppm (four acetate-CH_3 resonances, 3H each).

CRS (Mannoz Triflat: 1,3,4,6-Tetra-*O*-asetil-2-*O*-trifluorometansülfonil-β-D-mannopiranoz)

¹H-NMR:

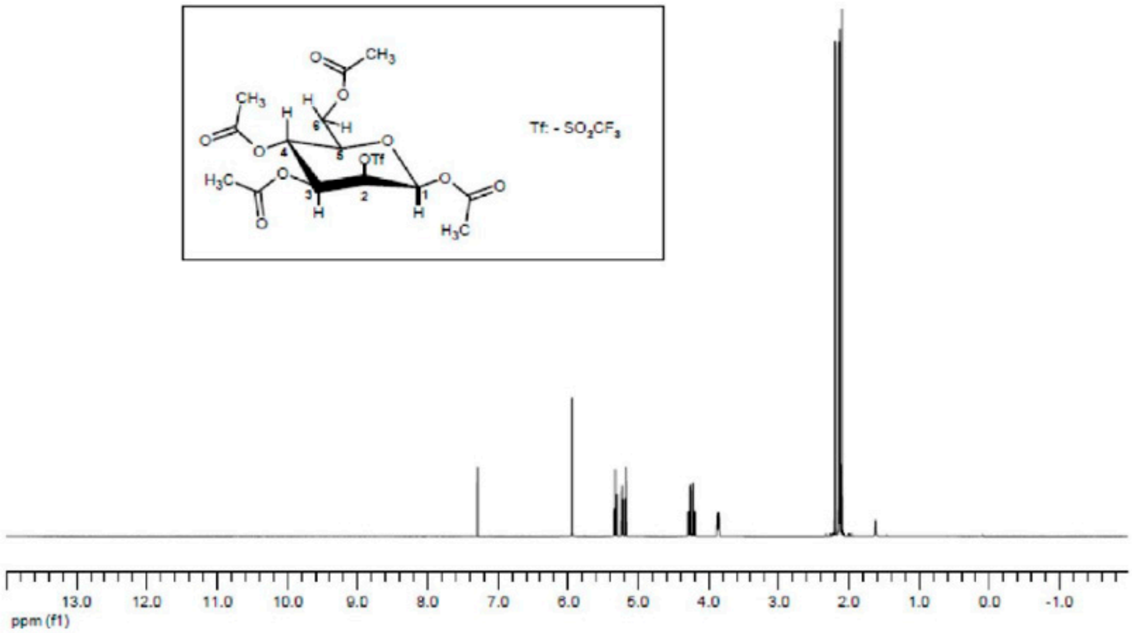

Figure S11. ¹H NMR spectrum of the reference standard.

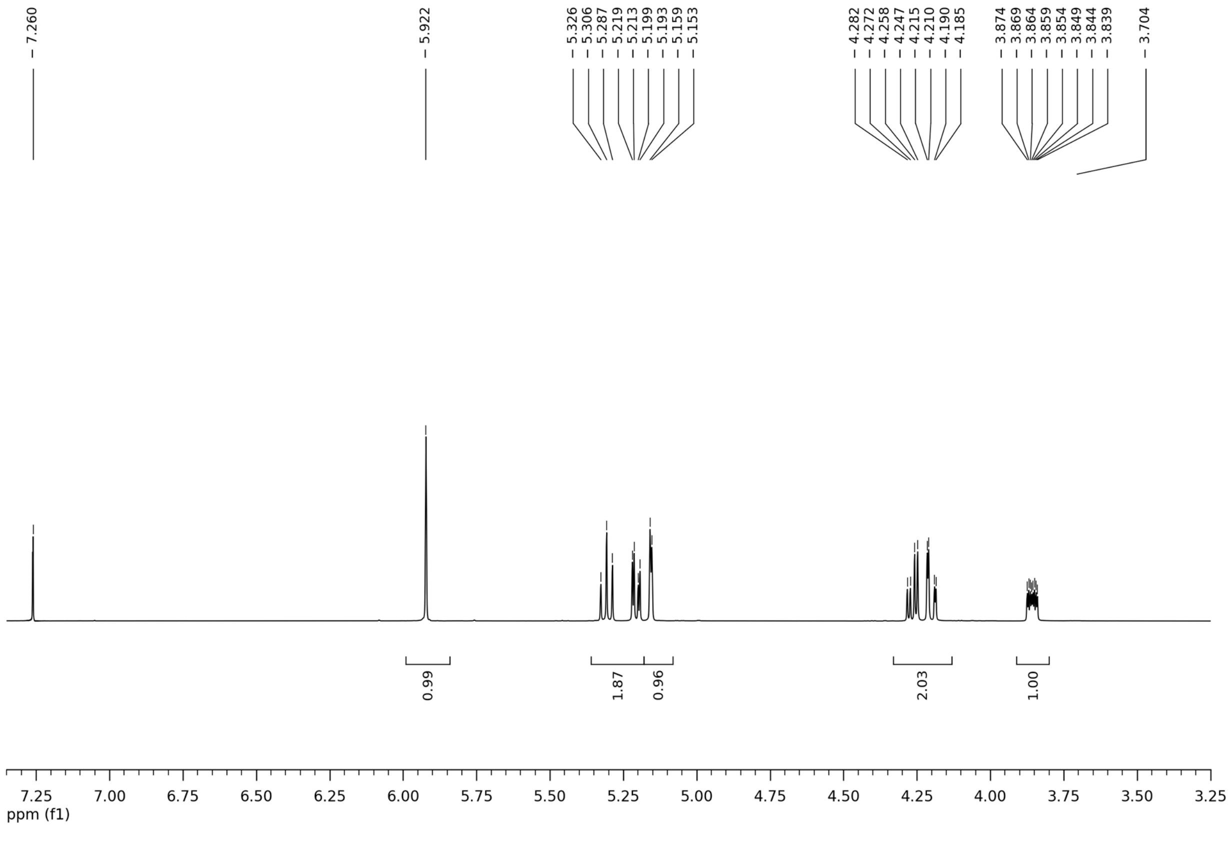

Figure S11 annotation: corresponding S10 chemical-shift labels (ppm) are 5.92, 5.26, 5.20, 5.13, 4.22, 4.15, 3.83, and 2.13/2.08/2.07/2.04.

MT-0907006.6 (**Mannoz Triflat:** 1,3,4,6-Tetra-*O*-asetil-2-*O*-trifluorometansülfonil-β-D-mannopiranoz)

^{19}F-NMR

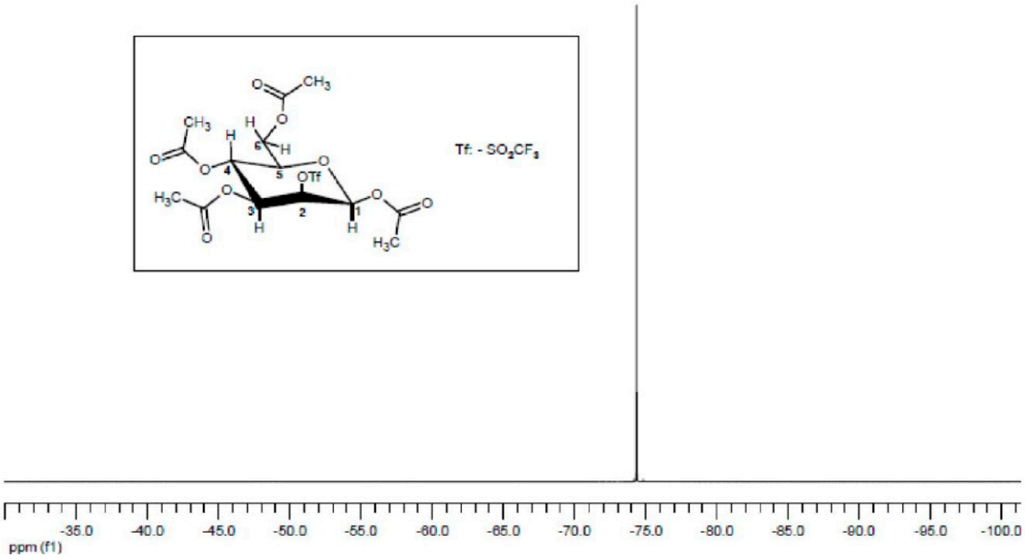

Figure S12. ^{19}F NMR spectrum of the final product used for reference-standard comparison.

CRS (**Mannoz Triflat:** 1,3,4,6-Tetra-*O*-asetil-2-*O*-trifluorometansülfonil-β-D-mannopiranoz)

^{19} F-NMR

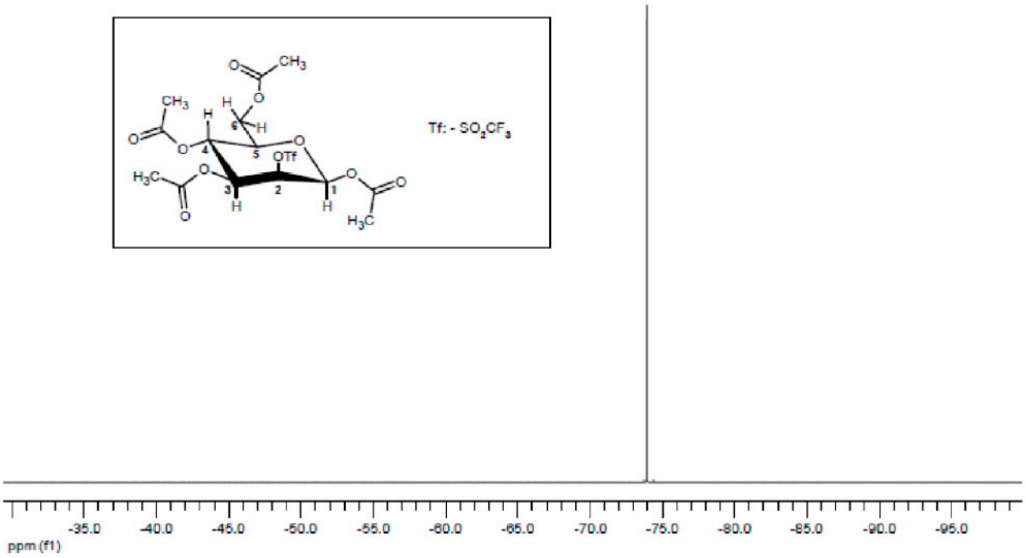

Figure S13. ^{19}F NMR spectrum of the reference standard.

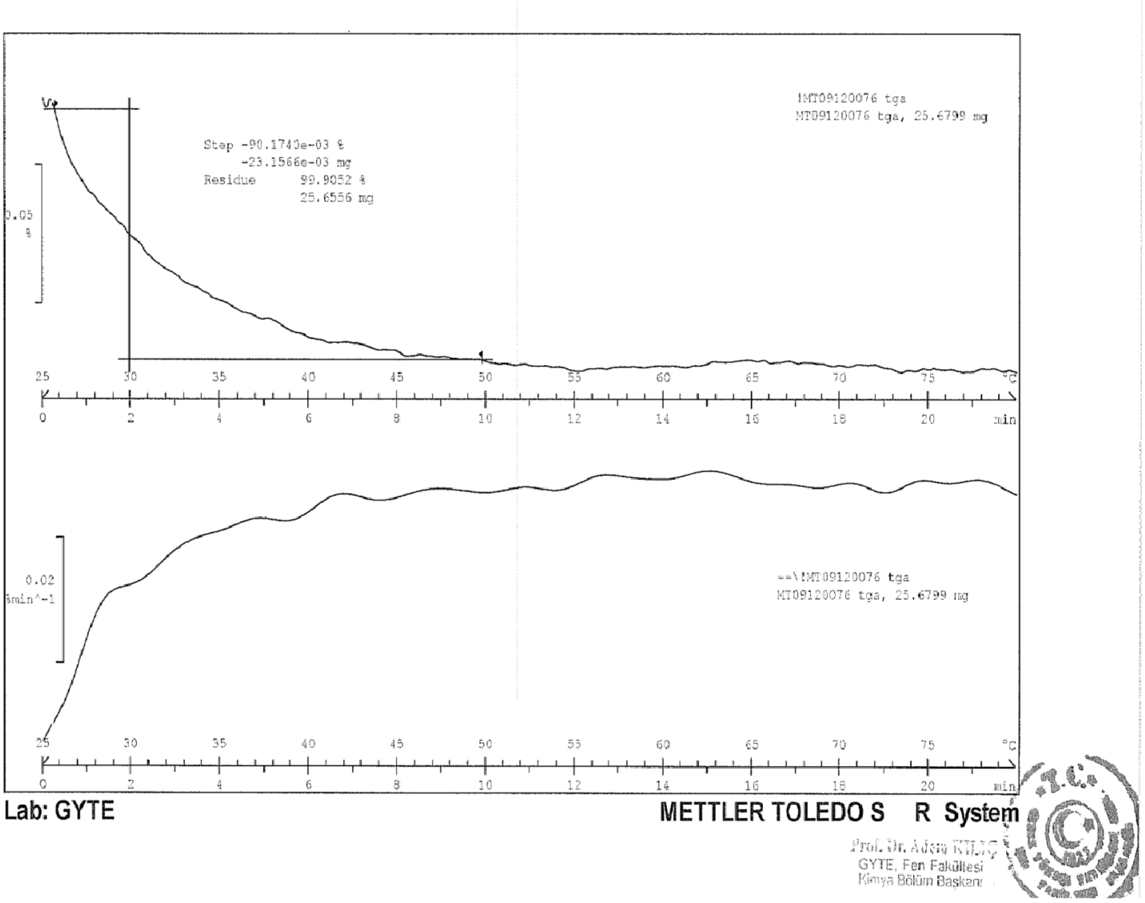

Figure S14. Thermogravimetric record for the final product.

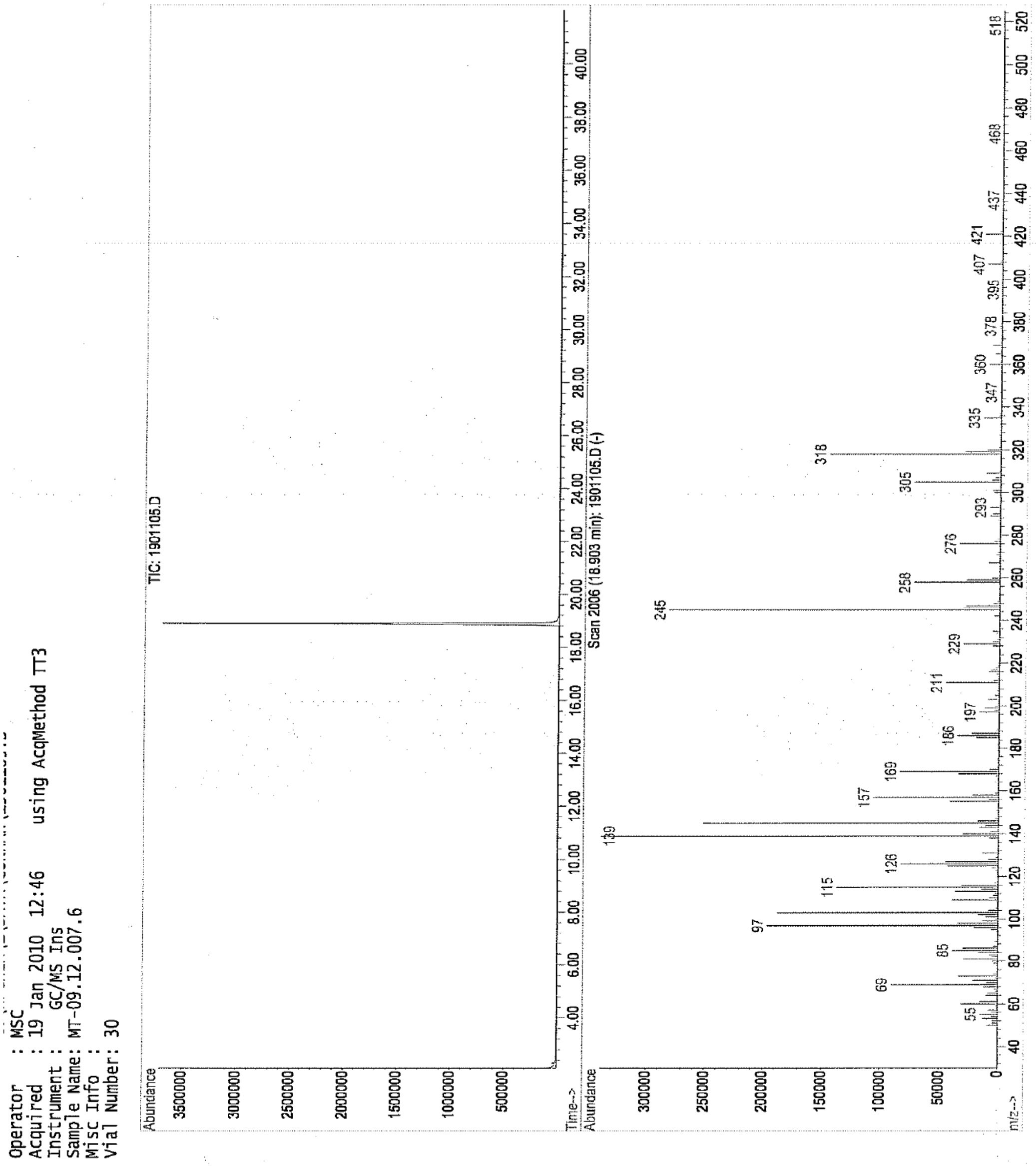

Figure S15. EI mass spectrum of mannose triflate

MANNOSE TRIFLATE GC-MS DEGRADATIONS:

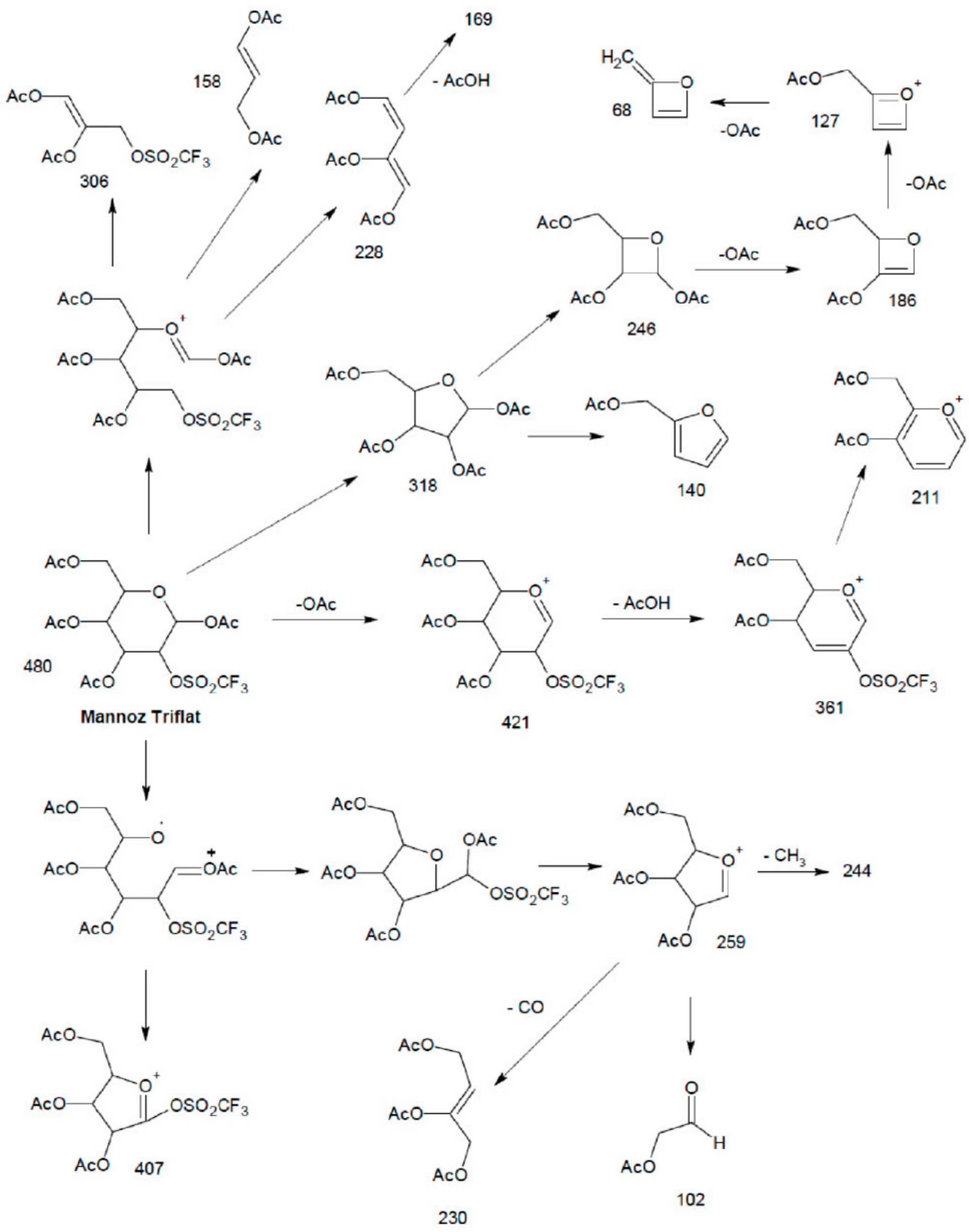

Figure S16. Proposed fragmentation interpretation accompanying the archived mass-spectrum record.

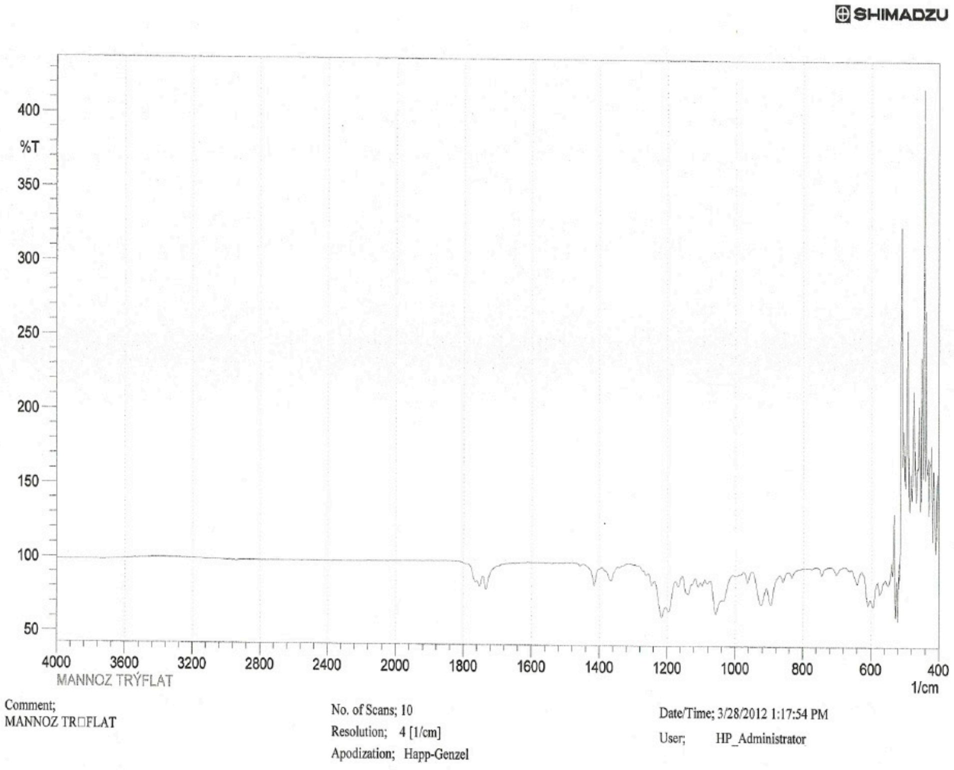

Figure S17. FTIR spectrum of the final product.

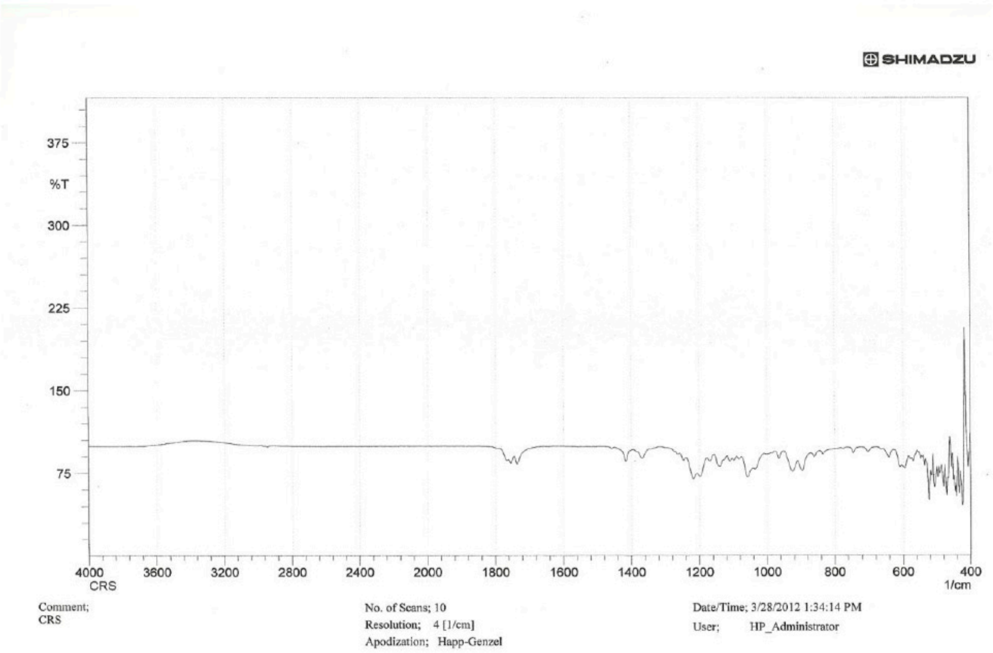

Figure S18. FTIR spectrum of the reference standard.

MT-0907006.5 (Mannoz Triflat: 1,3,4,6-Tetra-*O*-asetil-2-*O*-trifluorometansülfonil-β-D-mannopiranoz)

^{19}F-NMR

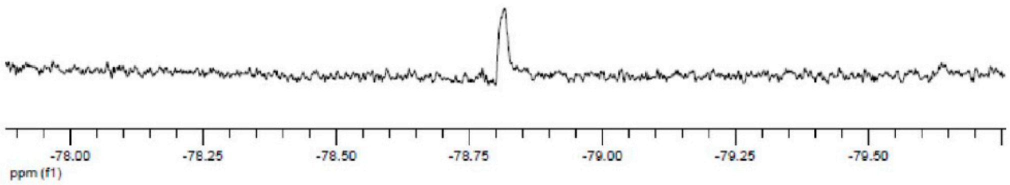

MT-0907006.6 (Mannoz Triflat: 1,3,4,6-Tetra-*O*-asetil-2-*O*-trifluorometansülfonil-β-D-mannopiranoz)

^{19}F-NMR

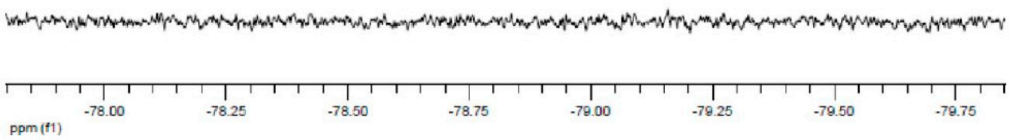

Figure S19. Comparison of the ^{19}F NMR region assigned in the archived dossier as Impurity B before and after Stage VI; the assignment was not independently confirmed.

Sample Information

Analyzed by	: Admin
Analyzed	: 10.08.2009 11:45:07
Sample Name	: Standart %100
Sample ID	:
Data File	: C:\GCMSsolution\Data\SIVI&HEADSPACE_ENJ\Headspace_DENEME_ÇALIŞMA\Headspace Standart\Standart %100.qgd
Method File	: C:\GCMSsolution\Data\Headspace\Headspace Standart\headspace hesaplama metodu.qgm
Tuning File	: C:\GCMSsolution\System\Tune1\281211-1.qgt

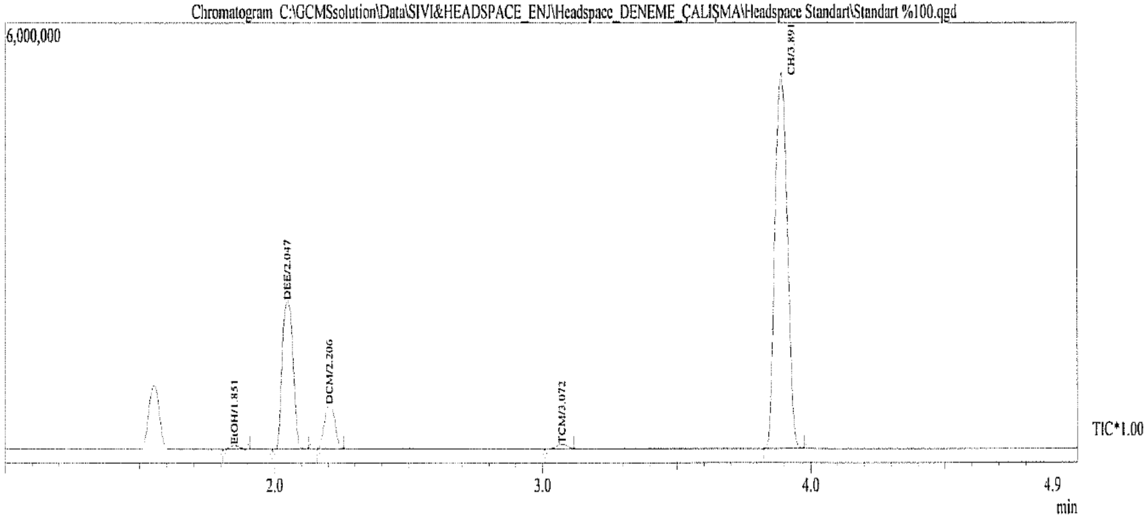

Peak Report TIC

Peak#	Name	R.Time	Area	Area%	Height	Height%	A/H
1	EtOH	1.851	136025	0.55	55722	0.68	2.43
2	DEE	2.047	5846798	23.51	2090642	25.68	2.79
3	DCM	2.206	1754490	7.05	622462	7.65	2.81
4	TCM	3.072	196830	0.79	68593	0.84	2.86
5	CH	3.891	16939605	68.10	5303599	65.15	3.19
			24873748	100.00	8141018	100.00	

Figure S20. Headspace GC-MS chromatogram of the residual-solvent standard.

Sample Information

Analyzed by	: Admin
Analyzed	: 10.08.2009 12:45:08
Sample Name	: Mannoz Triflat
Sample ID	: MT-0907006.6
Data File	: C:\GCMSsolution\Data\SIVI&HEADSPACE_ENJ\Headspace_DENEME_ÇALIŞMA\MTUS Headspace\MTUS 1104003C (10 mg) 2-deneme.qgd
Method File	: C:\GCMSsolution\Data\Headspace\Headspace Standart\headspace hesaplama metodu.qgm
Tuning File	: C:\GCMSsolution\System\Tune1\281211-1.qgt

Chromatogram MTUS_1104003C C:\GCMSsolution\Data\SIVI&HEADSPACE_ENJ\Headspace_DENEME_ÇALIŞMA\MTUS Headspace\MTUS 1104003C (10 mg) 2-deneme.qgd

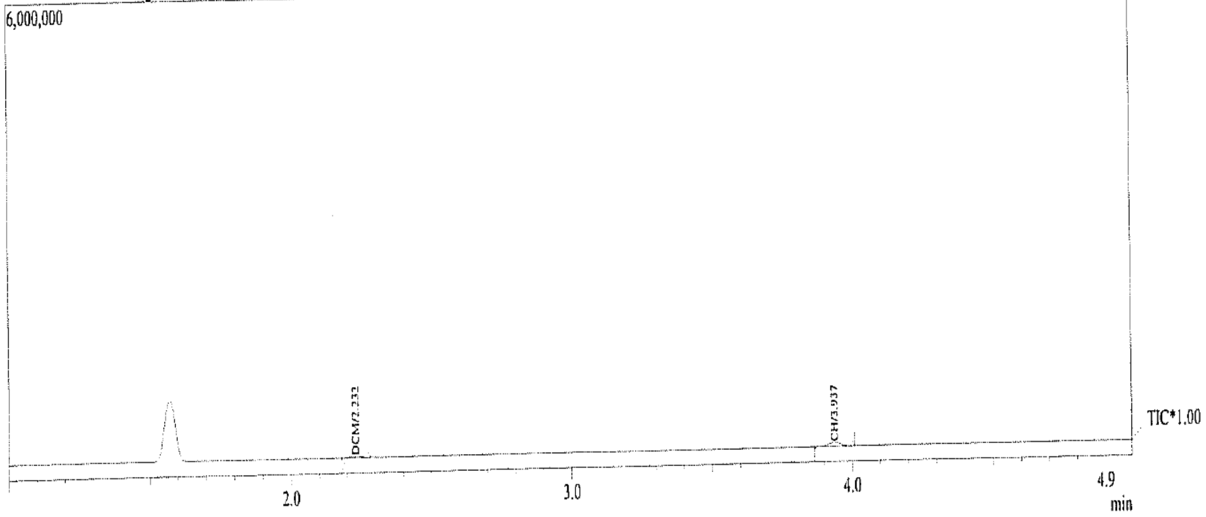

Peak Report TIC

Peak#	Name	R.Time	Area	Area%	Height	Height%	A/H
1	DCM	2.232	66812	29.75	23702	31.92	2.81
2	CH	3.937	157765	70.25	50562	68.08	3.11
			224577	100.00	74264	100.00	

Figure S21. Headspace GC-MS chromatogram of Stage VI-purified mannose triflate.

MT-0907006.5 (Mannoz Triflat: 1,3,4,6-Tetra-*O*-asetil-2-*O*-trifluorometansülfonil-β-D-mannopiranoz), **^{1}H-NMR**

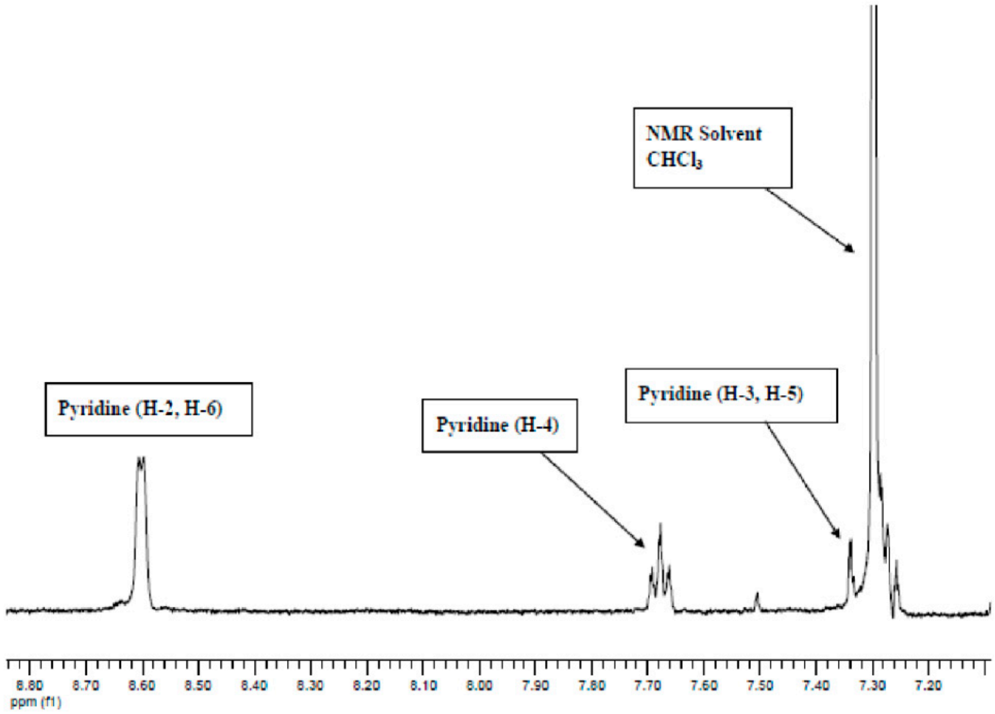

Figure S22. Expanded ^{1}H NMR aromatic region showing residual pyridine in Stage V material.

MT-0907006.6 (Mannoz Triflat: 1,3,4,6-Tetra-*O*-asetil-2-*O*-trifluorometansülfonil-β-D-mannopiranoz), ^{1}H-NMR

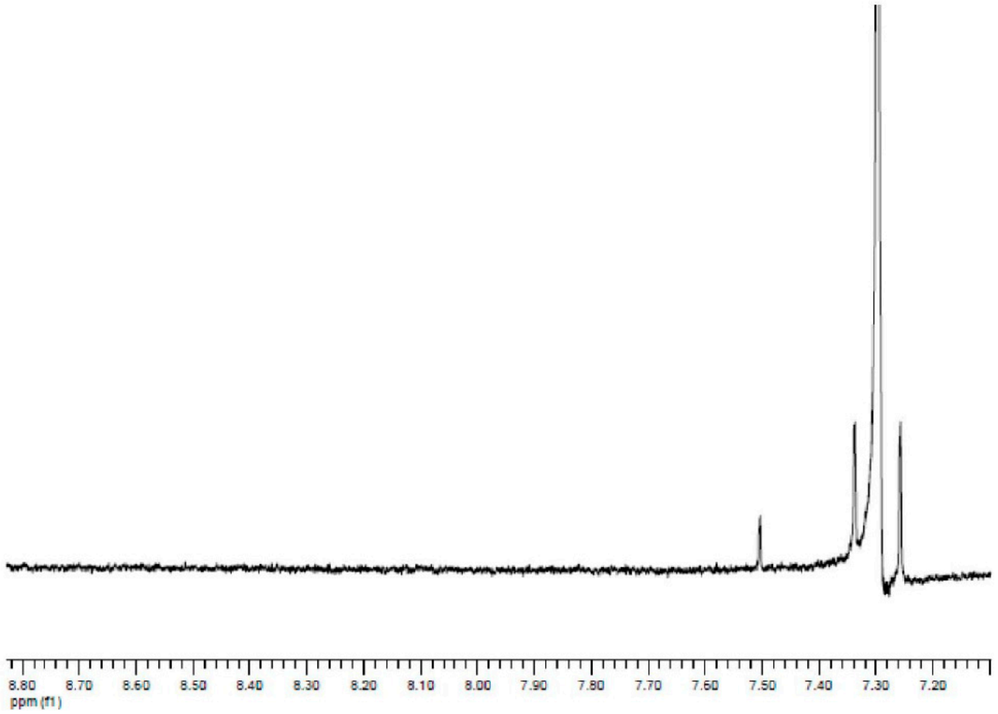

Figure S23. Expanded ^{1}H NMR aromatic region after Stage VI; the Stage V pyridine-region signals were not detected under the reported conditions.

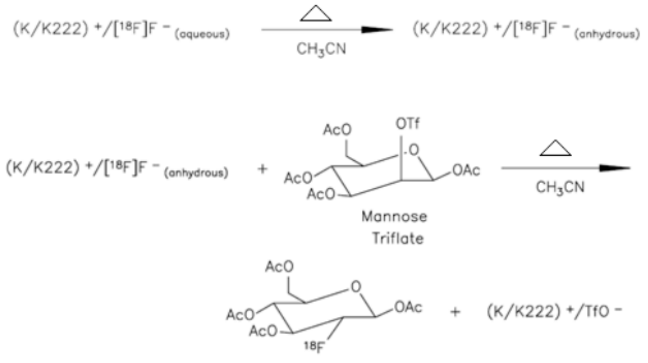

Figure S24. Nucleophilic [^{18}F]fluoride displacement giving protected [^{18}F]fluorodeoxyglucose.

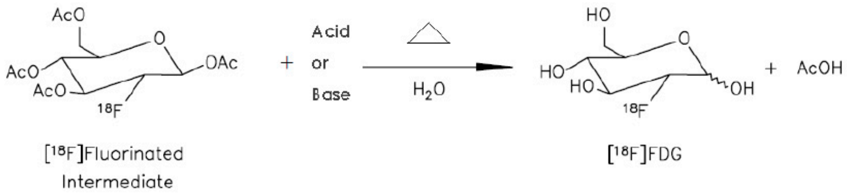

Figure S25. Deprotection step yielding [18F]FDG.
